# Influence of fluorophore and linker length on the localization and trafficking of fluorescent sterol probes

**DOI:** 10.1038/s41598-020-78085-9

**Published:** 2020-12-16

**Authors:** Jarmila Králová, Michal Jurášek, Lucie Mikšátková, Anna Marešová, Jan Fähnrich, Petra Cihlářová, Pavel Drašar, Petr Bartůněk, Vladimír Král

**Affiliations:** 1 CZ-OPENSCREEN, Institute of Molecular Genetics of the Czech Academy of Sciences, v.v.i., Vídeňská 1083, 142 20 Prague 4, Czech Republic; 2 University of Chemistry and Technology, Technická 5, 166 28 Prague 6, Czech Republic

**Keywords:** Biochemistry, Cell biology, Chemical biology

## Abstract

Fluorescent sterol probes, comprising a fluorophore connected to a sterol backbone by means of a linker, are promising tools for enabling high-resolution imaging of intracellular cholesterol. In this study, we evaluated how the size of the linker, site of its attachment and nature of the fluorophore, affect the localization and trafficking properties of fluorescent sterol probes. Varying lengths of linker using the same fluorophore affected cell penetration and retention in specific cell compartments. A C-4 linker was confirmed as optimal. Derivatives of heterocyclic sterol precursors attached with identical C-4 linker to different fluorophores at diverse positions also showed significant differences in their binding properties to various intracellular compartments and kinetics of trafficking. Two novel red-emitting probes with good cell permeability, fast intracellular labelling and slightly different distribution displayed very promising characteristics for sterol probes. These probes also strongly labelled endo/lysosomal compartment in cells with pharmacologically disrupted cholesterol transport, or with a genetic mutation of cholesterol transporting protein NPC1, that overlapped with filipin staining of cholesterol. Overall, the present study demonstrates that the physicochemical properties of the fluorophore/linker pairing determine the kinetics of uptake and distribution and subsequently influence the applicability of final probes.

## Introduction

The general approach to the design of fluorescent probes has been to attach a parent molecule with known binding selectivity and pharmacology to a fluorophore reporter. An ideal fluorescent molecule would retain the properties of the parent ligand. However, the addition of fluorophore to a known molecule alters its structure and may lead to changes in the pharmacology of the resulting molecule^1^. Therefore, the usefulness of such fluorescent probes has to be analyzed carefully in detail.

**Figure 1:**
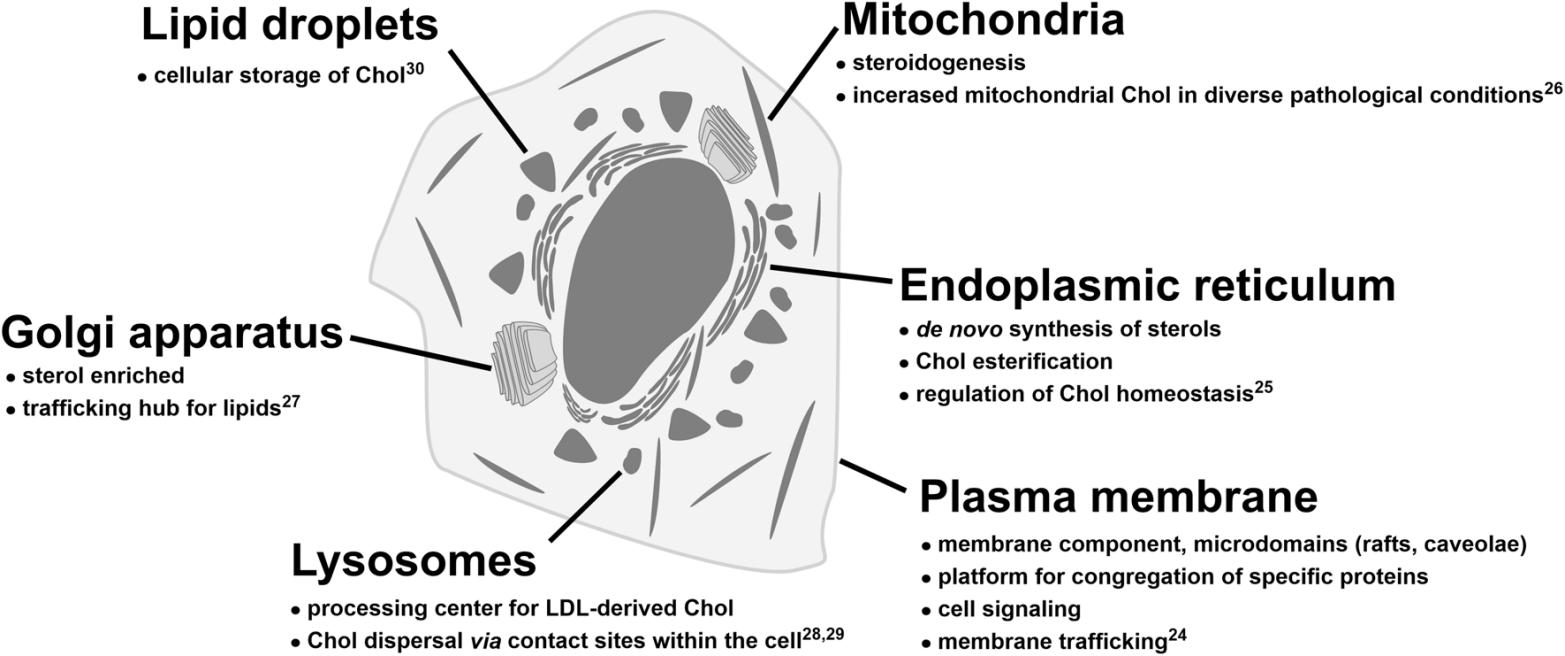
Compartmentalization of cellular cholesterol and its important functions with references to literature^24–30^.

Cholesterol (Chol) plays fundamental structural and functional role in membranes, particularly in the plasma membrane. More recently, it has become apparent that the presence of Chol in other intracellular compartments is also important (see Fig. 1). However, some developed probes for Chol visualization are not able to detect it in these compartments. It is likely that the sub-optimal properties of these probes may prevent the detection of lower levels of sterol^2^ or its esterified form, as is the case of filipin^3^. Therefore, reliable probes for Chol monitoring are still in demand^4–7^.

In our previous study^8^ we reported fluorescent probes based on heterocyclic sterol derivatives with various fluorophores attached to different regions on a sterol scaffold and revealed striking differences in their efficacy. A very promising probe was a conjugate of the green-emitting BODIPY fluorophore with sterol P1, designated as FP-5. This probe showed high cholesterol- and cholesteryl acetate-binding specificity in spectroscopic studies and fast labelling of cholesterol-rich compartments in cellular studies. Besides an ability to monitor the dynamics of sterol transport and intracellular trafficking in U-2 OS cells, it also demonstrated a pathological cholesterol accumulation in fibroblasts containing different mutations in the cholesterol transporter NPC1^8^. As a continuation of this research, we prepared FP-5 analogues with varying lengths of linker, extended the range of fluorophores, including green- and red-emitting ones, attached to sterol precursors on diverse rings. The aim of this study was to select a suitable fluorophore, locate the right linker position on a sterol scaffold and find an optimal linker length, all in order to obtain a high affinity sterol probe(s). Through the systematic and targeted design of fluorescently labelled sterol-like molecules, we have developed probes that are able to detect Chol in various intracellular compartments.

## Results

**Figure 2:**
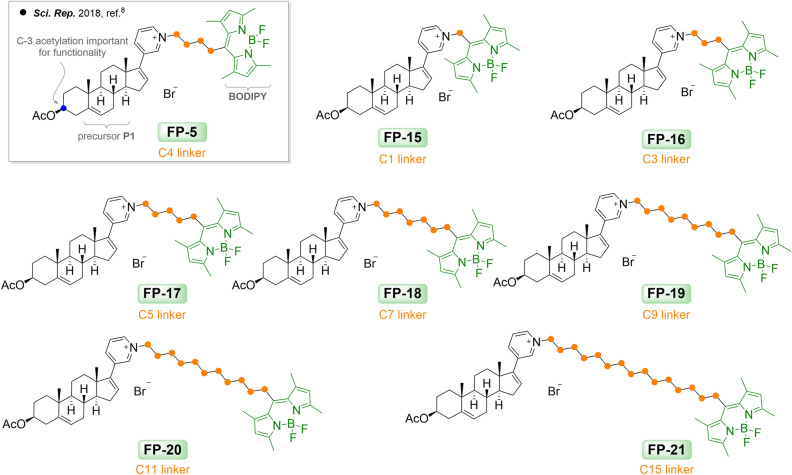
Probes FP-5 and FP-15–FP-21 with shortened and extended length of side chain.

We previously demonstrated the utility of the green-excited fluorophore BODIPY-abiraterone acetate, FP-5^8^. This derivative displayed good indices, which are required for an effective sterol probe. BODIPY green fluorophore was in this case attached on a pyridyl group via a C-4 linker (Fig. 2, Supplementary Fig. S2)*.* The acetyl group at the C-3 position was shown to be important for rapid uptake and trafficking in the cellular environment (depicted in Fig. 2 in grey rectangle). At the same time, we have shown that the gradual loss of the 3-*O*-acetate moiety resulted in the presence of both acetylated and deacetylated forms in the biological environment within 24 h. Unlike filipin, where the scientific community accepts that it only reacts with unesterified cholesterol, FP-5 probe appears to be associated with both entities, i.e. cholesterol and cholesterol esters.

In order to systematically test the effect of linker length on pharmacology of the final product, we synthesized a number of derivatives using the same sterol precursor P1 (Supplementary Fig. S1B), green BODIPY fluorophore and site of attachment, but varied the linker length (Supplementary Fig. S3A).

### Probes with varying linker lengths

The precursor P1 and synthetic route used for preparation of probes are shown in Supplementary Figures S4 and S5. Synthetic details of bromo-BODIPY fluorophores and “P1-linker-BODIPY” conjugates (probes FP-15–FP-21) are available as Supplementary Information, Sect. 2. The final probes FP-15–FP-21 with different length of linkers, i.e. C-1, C-3, C-4, C-5, C-7, C-9, C-11, and C-15 atoms, are summarized in Fig. 2.

**Figure 3:**
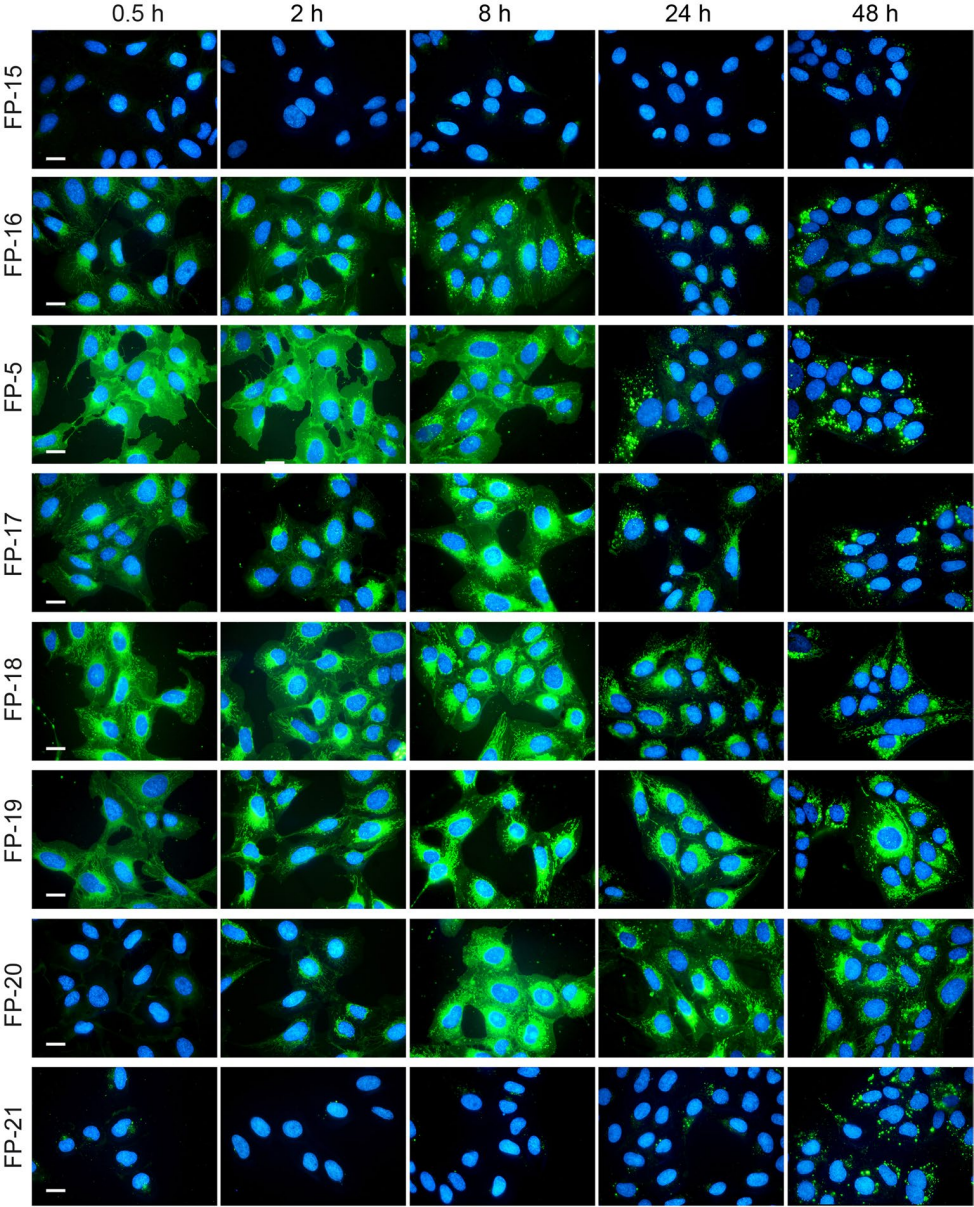
Uptake of FP-5 analogues by U-2 OS cells. FP-5 analogues at 200 nM concentration were added to the cultivation medium containing 5% LPDS and representative images of the localization of fluorescent probes in cells were taken at indicated times 0.5, 2, 8, 24, and 48 h. Nuclei labelled with Hoechst 33342 are shown in blue. Scale bar 10 μm.

Effectiveness of these probes was evaluated in cellular studies using U-2 OS cells. The previously described probe FP-5^8^ was included for comparison. Probes were added to cultivation medium containing lipoprotein-deficient serum (5% LPDS) and fluorescence was recorded at various time points after (0.5 h, 2 h, 8 h, 24 h and 48 h) (Fig. 3). Probe FP-5 with linker C-4 displayed quick cell penetrating fluorescence associated with the plasma membrane and intracellular compartments shortly after addition (within 0.5 h). However, probes either with short linker C-1 and C-3 (probes FP-15 and FP-16, respectively) or long linker C-11 and C-15 (FP-20 and FP-21, respectively) labelled cells under the same condition less and slower. Notably, when the same probes were administrated to cells in serum free medium, the fluorescence signal appeared or amplified within 20–24 h (see Supplementary Figs. S9 and S12). Probes FP-17, FP-18, and FP-19 with linkers C-5, C-7, and C-9 respectively, labelled cells similarly to FP-5, but with different accumulation. While FP-5, and also probes FP-15 and FP-16, within 2–24 h exhibited increasing signal in lysosomes and lipid droplets, as shown by co-localization with LysoTracker Red and LipiRed markers (see Supplementary Fig. S9), the fluorescence signal of FP-17–FP-21 stayed from the beginning associated mainly with ER and mitochondria without any later significant redistribution (Supplementary Figs. S10, S11, and S12).

The binding ability of probes to cholesterol was tested by UV–Vis spectroscopy in aqueous medium (H_2_O-DMSO; 9/1, *v*/*v*, resp.; Supplementary Fig. S7). The stability constants are summarized in Supplementary Table S3A. High stability constants were obtained for both 1:1 and 2:1 FP-5: cholesterol complexes, i.e. 6.0659 and 10.5986, respectively. Slightly lower but almost even values ranging from 5 to 7 were found for complexes FP-15, FP-17, FP-18, FP-19, and FP-20 with cholesterol. Probes FP-16 and FP-21 gave a relatively low value for the 1:1 complex.

Overall, these data indicate that the length of linker affects binding of probes to cholesterol as well as their uptake and distribution in living cells. A schematic summarizing the distribution of probes is shown in Supplementary Fig. S15.

### Probes with different fluorophores

**Figure 4:**
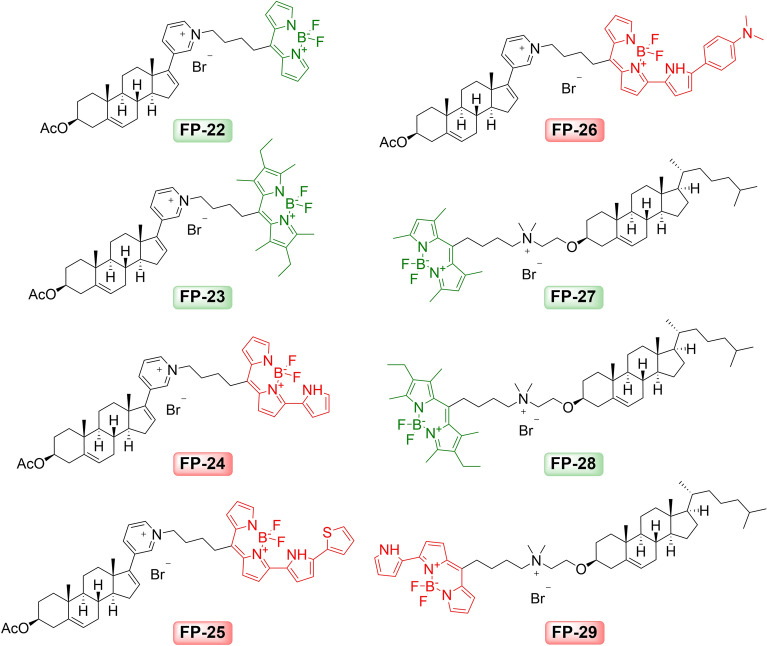
Sterol probes FP-22–FP-29 with various BODIPY dyes derived from P1 and P2 precursors.

Synthetic precursors P1 described in the previous report^8^ and newly tested precursor P2 (cholest-5-en-3β-oxyethan-*N*,*N*-dimethylamine) described by Bajaj^9^ (Supplementary Fig. S1B) were used in this investigation. Similar to what was described above, various bromalkyl BODIPY dyes (Supplementary Fig. S3B) have been used, but with different BODIPY modifications than the analogs shown in Fig. 2. These dyes with the same C4 linker (BODIPY s12-s16) were attached to precursors to form fluorescent probes FP-22–FP-29 (Fig. 4).

Probes FP-22 ‒ FP-26 have fluorophores attached via C17-pyridyl group of precursor P1 forming quaternary pyridinium salts, while probes FP-27 ‒ FP-29 derived from precursor P2 (Fig. 4 and Supplement, Fig. S1B) yielded “P2-dimethyl(BODIPY)ammonium” salts.

**Figure 5:**
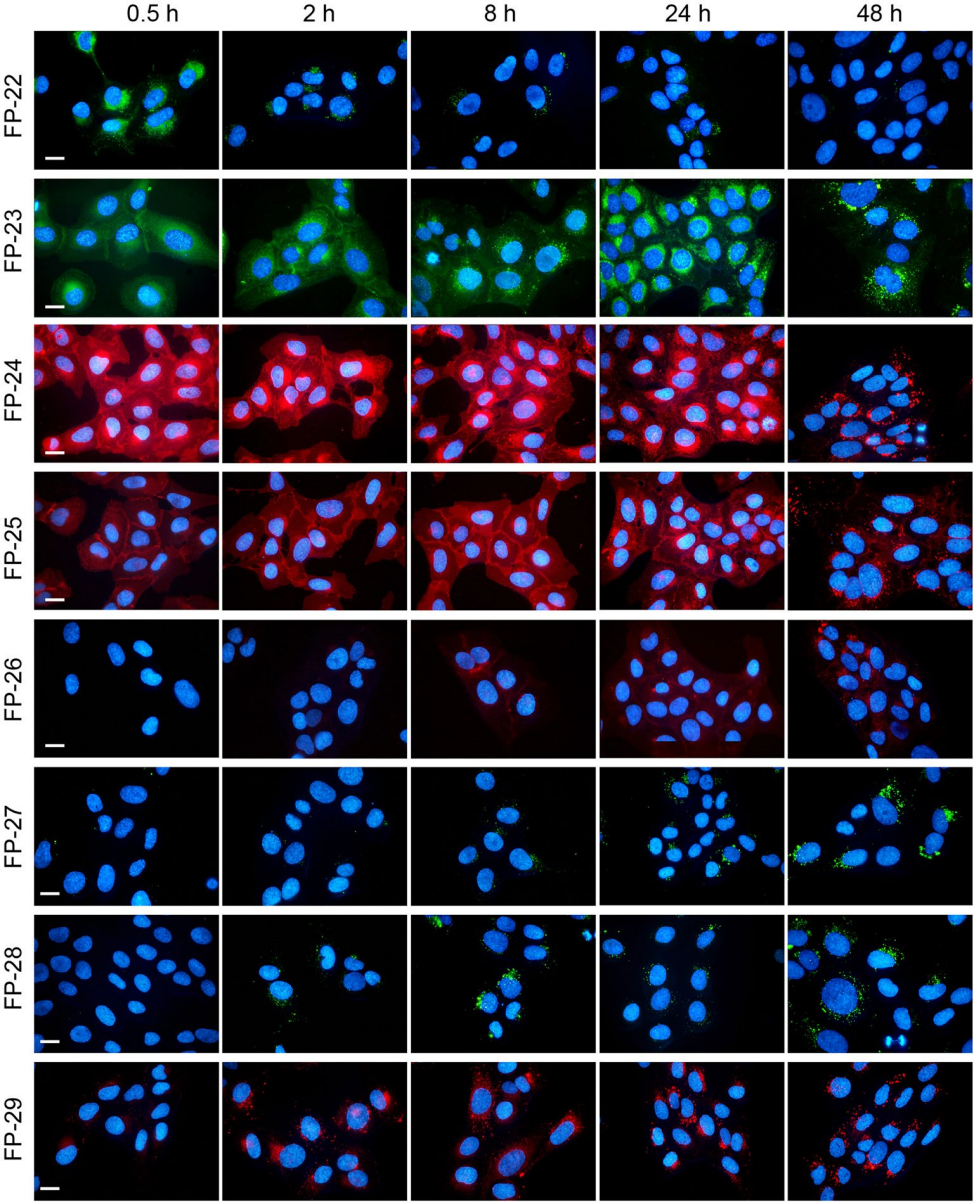
Cellular uptake and distribution of probes FP-22‒FP-29. Cells were loaded with 200 nM probes and representative images of the localization of fluorescent probes in cells were taken at indicated times 0.5, 2, 8, 24, and 48 h. Nuclei stained with Hoechst 33342 are blue. Scale bar 10 μm.

The cellular uptake, kinetics and distribution of these probes were investigated in U-2 OS cells. Cells were incubated in the presence of probes directly added to medium supplemented with 5% LPDS and photographed alive at the indicated time points under identical camera settings (Fig. 5). Probes FP-24 and FP-25, with red emitting fluorophores attached on the pyridyl group on the D ring, provided a strong and stable signal with slightly different kinetics and distribution. Probe FP-24 entered cells quickly and spread within 0.5 h from the plasma membrane to the cytoplasm, ER, Golgi apparatus, and mitochondria (Supplement Fig. S13). Later (2–8 h) there was a notable accumulation of probe fluorescence in lysosomes and within 24–48 h also in lipid droplets (LD) (Supplement Fig. S13). Probe FP-25 entered cells with slower kinetics and labelled mainly the plasma membrane, cytoplasm and slightly ER and Golgi apparatus. Its moderate redistribution to lysosomes and LD was noticeable within 24–48 h after addition (Supplement Fig. S14). In contrast, FP-27‒FP-29 probes, with fluorophores attached on the A ring, labelled cells poorly (Fig. 5).

The potential of FP-24 and FP-25 probes to bind cholesterol and cholesterol-3-*O*- acetate was tested by UV/VIS spectroscopy (Supplementary Fig. S8). Relatively high stability constants of both probes with cholesterol confirmed this potential (Supplementary Table S3B). Notably, probe FP-25 displayed a low *K*s value (1.33) for 1:1 complexes with cholesterol acetate (Supplement Table S3B).

**Figure 6:**
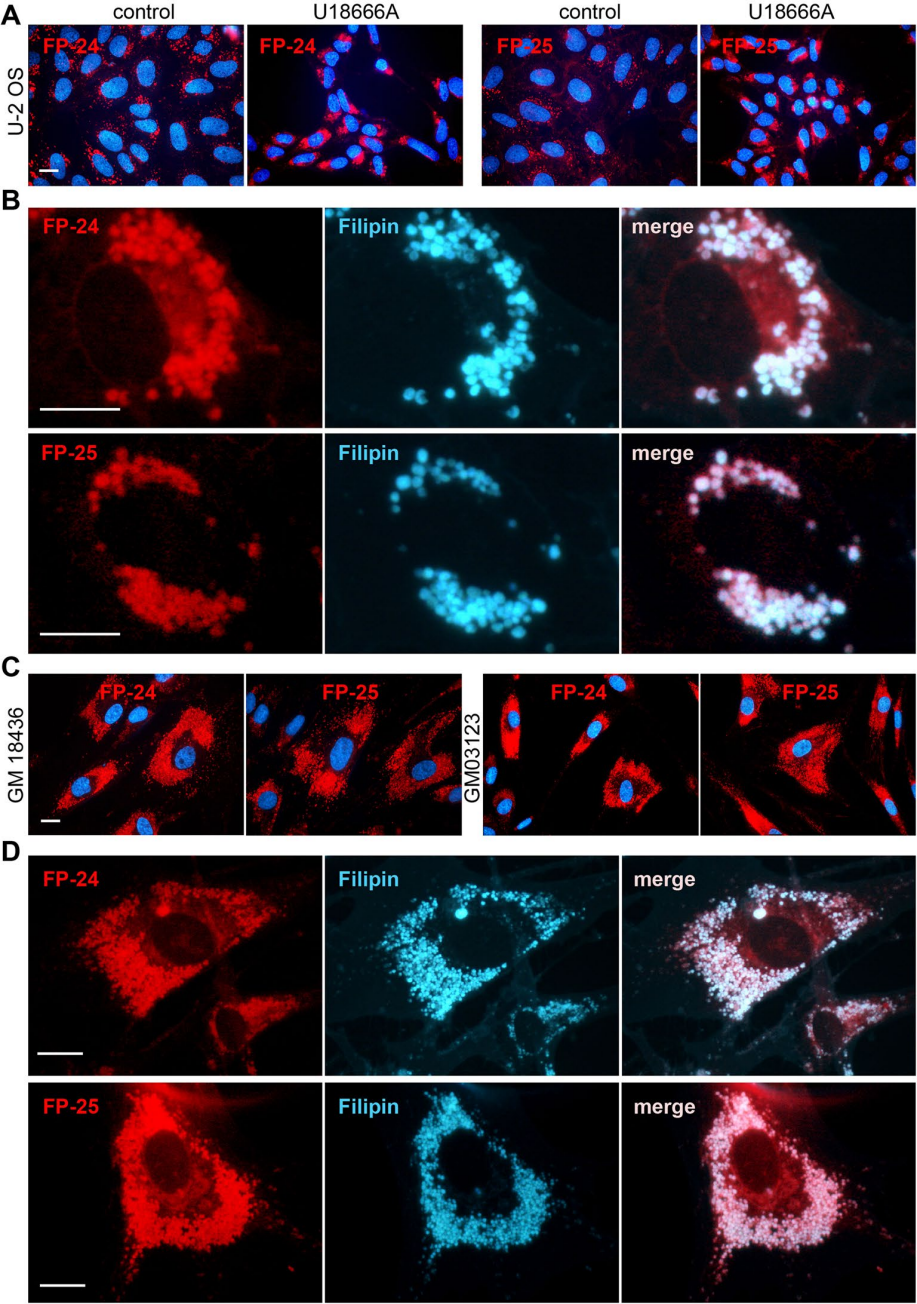
FP-24 and FP-25 fluorescence in cells with abnormal content of cholesterol. (**A**) Cholesterol transport in U-2 OS was inhibited by inhibitor U18666A (1 μg/mL) for 48 h, then cells were labelled with indicated probes (100–200 nM) for an additional 24 h and examined. Control cells were treated with vehicle only. (**B**) Co-localization of FP-24 and FP-25 staining with filipin. U-2 OS cells were treated with inhibitor, labelled with probes, fixed and stained with filipin (50 μg/mL). About 70% of cells displayed significant co-localization of filipin with probe FP-24 (evaluated 107 cells) and 60% with probe FP-25 (evaluated 100 cells). (**C**) Human fibroblasts carrying mutations in NPC1 cholesterol transporter (clones GM03123E, GM18436) were labelled with probes for 20 h and examined. (**D**) Co-localization of FP-5 and filipin staining in mutant cell clone GM18436. About 90% of cells displayed significant co-localization of filipin with probe FP-24 (evaluated 70 cells) and 82% with probe FP-25 (evaluated 60 cells). Scale bars 10 μm.

Importantly, both probes strongly labelled the endo/lysosomal compartment of cells treated with the inhibitor of cholesterol transport, U18666A (Fig. 6A). Their staining co-localized with cholesterol binding filipin III (Fig. 6B). Also, human fibroblasts bearing different genetic mutations (clones GM18436 and GM03123) in the cholesterol transporter NPC1 were extensively labelled with both probes (Fig. 6C) at the same location as filipin (Fig. 6D). These results confirm the specificity of binding of these probes to places with accumulated cholesterol.

## Discussion

The efficacy of fluorescent probes depends on several factors^10^. To uncover the important structural determinants contributing to the improvement of effective sterol probes, we have prepared different types of derivatives. Firstly, we prepared derivatives based on the promising structure of the previously discovered FP-5 probe^8^ and varied the linker length. Shortening of the linker to C-1 in probe FP-15, resulted in poor cellular fluorescence (Fig. 3), which did not match with the relatively high stability constant when complexed with cholesterol (Supplementary Table S3A). However, when the same probe was administrated in serum free medium, the signal increased (Supplementary Fig. S9). This may indicate that this particular derivative interacts with some serum component(s), preventing the resulting complex from being effectively taken up by cells. An alternative explanation may be that the probe binds to some intracellular target and, as a result of this binding, the photophysical behaviour of the probe significantly changes and fluorescence is silenced as described for protein-targeted sensors^11^. In addition, a relatively low value of lipophilicity of the FP-15 probe (Supplementary Table S1) is likely contributing to its low efficacy, as we previously reported the impact of probe lipophilicity on cellular uptake^8^.

FP-16, FP-5 and FP-17 probes with medium sized linkers (C-3, C-4, and C-5, respectively), entered cells easily and their localization changed over time. It appeared on the plasma membrane as well as in intracellular membranes; temporarily associated with ER and mitochondria (within 30 min), later with lysosomes (within 2–24 h) and eventually was strongly accumulated in lipid droplets (Supplementary Figs. S9–S11).

In contrast, probes FP-18 and FP-19, with extended linkers C-7 and C-9, showed strong fluorescence mainly in the endoplasmic reticulum (ER) and mitochondria (Supplementary Fig. S10B). The localization of these probes did not significantly change over 24 h (Supplementary Figs. S10C and S11). There is a correlation between the lengthening of linker length and the calculated lipophilicity of these probes (Supplementary Table S1). Compounds with the nature of lipophilic cations are known to accumulate in the mitochondria^12,13^, therefore it is not surprising that probes with longer linkers and higher lipophilicity tends to be retained in the negatively charged mitochondrial matrix without significant re-localization. However, when the linker size was extended to C-15 in probe FP-21, there was a dramatic reduction in probe entry into cells, probably because of the size of the molecule as well as the low stability constant for 1:1 probe:cholesterol complex (Figs. 2, 3; Supplementary Table S3). However, when FP-21 was administered to cells in serum free medium, a signal associated with ER/mitochondria was detected (Fig. S12).

A similar observation that the length of linker strongly affects cellular localization of probes was demonstrated in the current O’Connor work^14^. This study reported two identical BODIPY derivatives that differ only in the length of the aliphatic chain between the dye and cholesterol. The probe directly ester linked to cholesterol partitioned strongly into the liquid-disordered (L_d_) phase, while the insertion of a hexyl linker between the probe and cholesterol directed the conjugate into a liquid ordered (L_o_) phase of giant unilamellar vesicles (GUVs). The profound impact of the linker length on uptake and distribution was also demonstrated at the living cell membrane. Conjugate without linker permeated the membrane and localized strongly to lipid droplets, whereas probe with a C-6 linker was retained at the living cell membrane, associated with sterol-rich regions. In contrast, our probes with longer linkers were mainly retained at the intracellular membranes. Using a steroid skeleton in combination with pyridinium salt to ensure the solubility of the conjugate may be a possible reason for a diverse membrane preference. In any case, our study confirms the general influence of linkers and shows, in addition, the importance of lipophilicty on the uptake and distribution of probes. A medium size of linker (C-4) seems to be optimal for following sterol uptake and trafficking.

Other important elements for probe efficiency are the structure, size and site of fluorophore attachment to the recognition part of probe. The conjugation of a fluorescent dye introduces a large amount of steric bulk to a known ligand, which can significantly alter the pharmacology and physicochemical properties of the resulting molecule^1,10^. Various substituted BODIPY scaffolds were synthesized in the past by various groups^15–20^. Our results confirmed that the alkylation or arylation of the BODIPY core, linked to sterol precursors by the same linker C-4, affects spectral properties as well as intracellular accumulation of the resulting probes. The non-methylated BODIPY probe FP-22 labels cells poorly (Figs. 4, 5). Probe FP-23, with a fully alkylated BODIPY core, displayed better efficacy but not as good as FP-5 probe with tetra-alkylated BODIPY (Figs. 4, 5). In this respect, a partially tetra-alkylated BODIPY core appears to be more beneficial than that completely alkylated.

Importantly, two new probes FP-24 and FP-25 implementing red-shifted BODIPY analogues provided strong fluorescence signal in cells. The introduction of extended conjugation at the α-position of the BODIPY moiety (Supplementary Fig. S1C, D) causes a significant red shift in excitation and emission wavelengths^21^. The corresponding BODIPY analog was reported to manifest decent fluorescence quantum yields, longer absorptions/emissions, insensitivity to the solvent polarity, and high biocompatibility due to structurally nearness to the natural red pigment prodigiosin^18^. In the case of our probe FP-24, the pyrrole moiety was conjugated to BODIPY fluorophore at the same α-position and the resulting probe exhibited accordingly absorption and emission maxima at 574 and 599 nm (Supplementary Fig. S6 and Table S2), fast cell penetration and dynamic intracellular distribution. Interestingly, the derivative with an attached phenyl group at the α-position, described in our previous study as probe FP-6 (Supplementary Fig. S2), labelled cells poorly^8^. This means that the position of the modifying group on the BODIPY core significantly affects not only the red-shift in fluorescence but also the cellular uptake of probe. When the fluorophore with pyrrole was further functionalized by a 2-thiophenyl group forming a thiophenyl-pyrrol BODIPY conjugate (Fig. 4), the corresponding probe FP-25 provided even longer red-shift fluorescence (λ_Amax_ = 618 nm and λ_Em_ = 676 nm; Supplementary Fig. S6 and Table S2) and a good cell signal associated mainly with cell membranes (Fig. 5). Moreover, the ability of FP-24 and FP-25 to label intracellular compartments efficiently in cells with accumulated cholesterol, caused either by treating cells with the inhibitor of cholesterol transport U18666A or in NPC1 mutants (Fig. 6), demonstrates the strong potential of these probes to monitor cholesterol. Therefore, they represent unique red-shifted probes with good specificity and efficacy.

Liu et al. previously described several red-shifted BODIPY-cholesterol conjugates, designated as s1, s2, and s3 (Supplement Fig. S2)^21^. In their case, aromatic substitutes (phenyl or thiophene or both) were attached to the BODIPY moiety in places other than our probes, so their structure is different. Besides, it took a MβCD carrier to bring them into the cells, while the advantage of our probes is that they enter cells effectively without the carrier.

In addition, we demonstrate that the probe efficacy is influenced also by the site of fluorophore attachment. Attaching the same fluorophores via the pyridyl group on the D ring of precursor P1 or on the A ring of precursor P2 (Figs. 4, 5; Supplementary Fig. S1B) resulted in a striking difference in cellular fluorescence. Specifically, the probes FP-23 and FP-24 gave significantly higher cell fluorescence than their respective counterparts FP-28 and FP-29 (Figs. 4, 5). In general, it can be said that probes FP-22‒FP-26 with fluorophores attached on D ring displayed a better fluorescence signal in cells than probes FP-27‒FP-29 with fluorophore linked to A ring, which is consistent with our previous report^8^.

**Figure 7:**
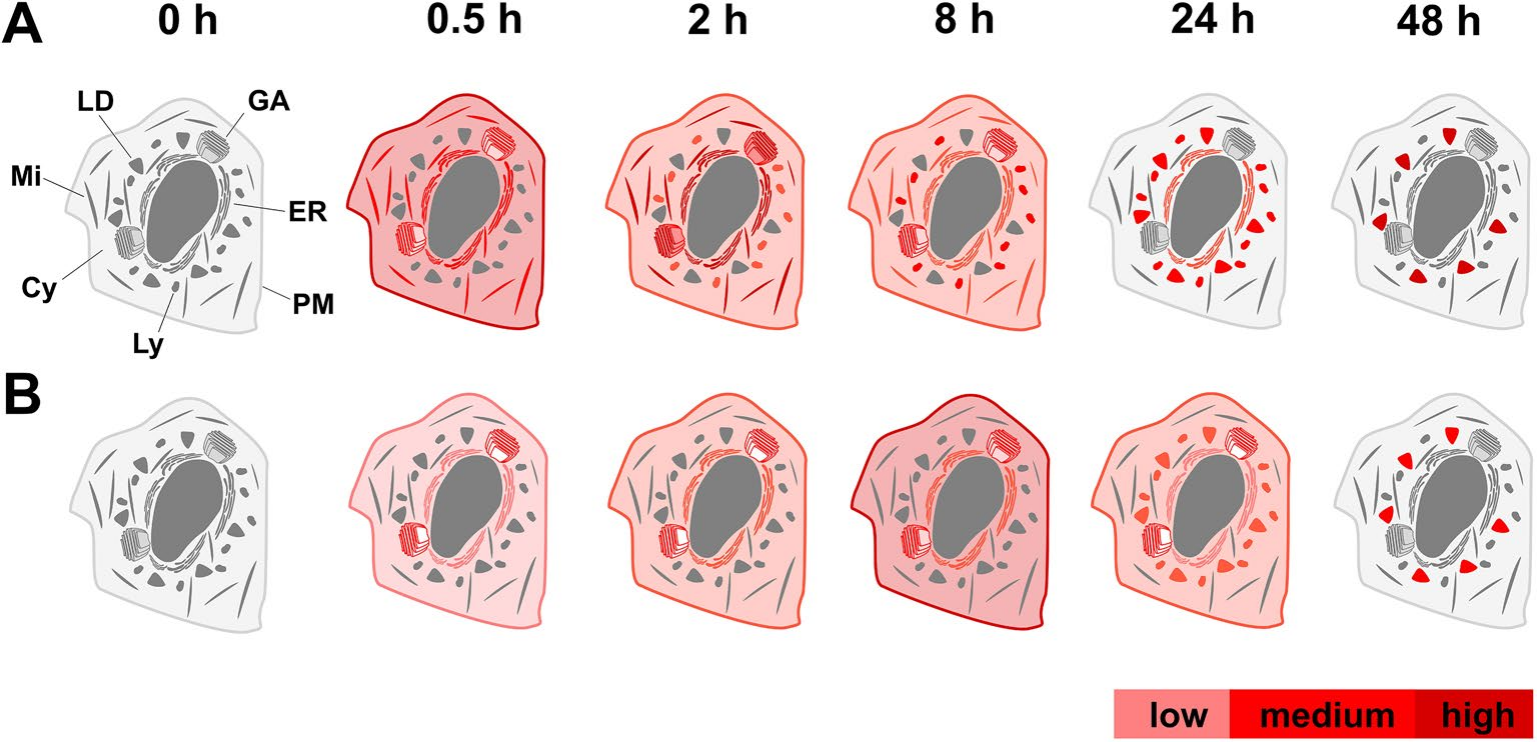
Schematic distribution of FP-24 (**A**) and FP-25 (**B**) signal in U2-OS cells.

In summary, the present study demonstrates that the design of fluorescent probes needs to take into account the complex influence of fluorophore, associated linker and site for fluorophore attachment. The efficacy and pharmacological properties of the final product are influenced further, among other things, by lipophilicity of its molecules and affinity to cell targets. It is therefore necessary to carefully test and select the most suitable designed structures for each specific case. Our systematic approach has led to the discovery of two very effective probes—FP-24 and FP-25, whose distribution is schematically shown in Fig. 7. Possessing red-shift emission, these two probes extend the repertoire of prior probes, which so far emit mainly in green channels.

## Materials and methods

### Reagents and materials

Precursor P1 (abiraterone acetate) was purchased from Steraloids. Solvents were purchased from PENTA, LysoTracker Red DND-99, LysoTracker Green DND-26 and MitoTracker Green FM, MitoTracker Deep Red FM, ER-Tracker Red (BODIPY TR Glibenclamide), ER-Tracker Blue-White DPX were from Molecular Probes (Life Technologies), Lipi-Green and Lipi-Red from Dojindo Molecular Technologies, CellLight Golgi-GFP from Thermo Fisher Scientific, LPDS (lipoprotein deficient serum) and Filipin III were from Sigma. Media RMPI, EMEM, FluoroBright DMEM, fetal bovine serum (FBS) and supplements were from Life Technologies. Inhibitor U-18666A was obtained from Enzo Life Sciences.

### Chemical synthesis and compound characterization

Synthesis of compounds and their characterization is included in Supplementary Information 2.

### Cell lines and cell culture

U-2 OS cells (obtained from ATCC) were cultivated in RPMI 1640 medium supplemented with 10% FBS, sodium pyruvate, 2 mM glutamine, penicillin and streptomycin (Sigma), 20 mM HEPES, and glucose (4 mg/mL)^8^. NPC1 fibroblasts (obtained from Coriell Repository) were maintained in EMEM medium supplemented with NEAA (nonessential amino acids), 2 mM glutamine, penicillin and streptomycin (Sigma), and 15% FBS as described previously^8^.

### UV–VIS and fluorescence spectra

Absorption and fluorescence spectra were measured in DMSO using quartz cells of 1 cm path length. Absorption spectra were recorded using Cary 60 spectrophotometer (data interval 0.5 nm, averaging time 0.1 s). For fluorescence measurement, a Cary Eclipse fluorescence spectrophotometer equipped with R3896 PMT was used (data interval 0.5 nm, averaging time 0.1 s, PMT voltage 600 V, excitation filter “Auto”, emission filter “Open”, slit width 2.5 nm both for excitation and emission, only for FP-22 and FP-25 both slits were widened to 10 nm). Absorption spectra were measured against air. Spectrum of solvent was recorded prior to addition of concentrated sample solution and was subtracted during data treatment in Microsoft Excel. Solution from the absorption cell was then further diluted with dimethyl sulfoxide in the fluorescence cell so that maximum absorbance of the solution in the fluorescence cell was lower than 0.1. Absorption spectrum of this solution was also measured. Fluorescence spectra of the solvent measured at the same conditions were subtracted from the fluorescence spectra of samples.

For correction of fluorescence spectra, correction curves supplied by the manufacturer for the range 220 to 600 nm were used. Emission correction curve was extrapolated to longer wavelengths using an incandescent lamp with coiled wire operated at several currents, which was considered to be approximately tungsten thermal radiator with single temperature. This temperature was evaluated from the wavelength range where the emission correction function is known.

Spectra show absorption coefficient calculated from the more concentrated solution measured in the absorption cell. For comparison, also the absorption coefficient calculated from absorption measurement of solution used for fluorescence measurement is plotted. Corrected excitation and emission curves are in relative unit-less values scaled to enable comparison with absorption curves. All of the spectra measured are shown in Supplementary Fig. S6 and the results are summarized in Table S2.

### UV–VIS titration

The stability constants of the probe with cholesterol and cholesteryl acetate were studied by UV–Vis spectroscopy in aqueous medium (H_2_O-DMSO; 9/1 (*v*/*v*). Solutions were prepared from DMSO stocks by dilution into buffer in conventional 1 cm PMMA cells. The concentration of the probe used was 0.3 μmol·L^−1^, and the concentration of the analytes varied from 0 to 47.6 µmol·L^−1^. Absorbance spectra of these solutions were recorded over wavelengths 300–700 nm using a GBC Cintra 404 spectrometer. Stability constants (*K*s) were calculated from absorbance changes in the probe using their maximum absorbance (ΔA) by nonlinear regression using online software Bindfit6. All of the spectra measured are shown in Supplementary Fig. S7 and S8 and the results are summarized in Table S3.

### Labelling of cells with fluorescent probes and organelle markers

Cells were plated and grown for one day in RPMI medium supplemented with FBS. Media was then changed to FluoroBright DMEM, either serum-free or containing 5% LPDS, for one hour before loading probes. Solutions of probes were prepared in DMSO and applied to cultivations (50–200 nM final concentration) for the indicated time points. Hoechst 33324 (final concentration 1 μM) was added into medium for the last 5 min of incubation to stain cell nuclei. For co-localization studies, cells were incubated with probes and subsequently loaded with organelle markers ER-Tracker Red (BODIPY TR Glibenclamide) (1 μM), or ER-Tracker Blue-White DPX (250 nM), LysoTracker Red DND-99 (50 nM), LysoTracker Green DND-26 (300 nM), MitoTracker Green FM (100 nM), MitoTracker Deep Red FM (80 nM), Lipi-Green (300–500 nM), Lipi-Red (1 μM) for 30 min at 37 °C. To avoid an artefact and erroneous interpretation caused by occasional red emission of green BODIPY in LDs^22^, we used a recommended protocol for doubly labelled samples^23^. The photographs of red fluorescence were collected first, then filter sets were changed for green fluorescence in the same field. The Golgi apparatus was tagged by adding CellLight Golgi-GFP reagent to the medium for 20 h and then co-stained with sterol probes for 1 h. For filipin co-staining, cells were first loaded with probes, washed with PBS, fixed with paraformaldehyde and then labelled with filipin for 30 min.

Experiments demonstrating fluorescence and localization have been performed independently at least three times for each probe. In each experiment, we collected fluorescence images of three to six regions comprising 10–25 cells (60–400 cells in total) under identical setting. The images shown are representative samples for each tested variant. For co-localization experiments, the percentage of cells was evaluated where the overlap of probe signal with filipin was visible at least on 50% of the cell area examined.

### U18666A treatment

U-2 OS cells were treated with 1 μg/mL U18666A for 40–48 h. Subsequently, cells were labelled with specified probes at final concentration 200 nM for an additional 16–24 h in FluoroBright medium with 5% LPDS and examined.

### Filipin co-staning

Cells either or not treated with U18666A were first loaded with specified probes, washed with PBS, fixed with 2% paraformaldehyde and then labelled in the dark with filipin (50 μg/mL) in PBS for 30 min. The fluorescence of probes (green or red) and filipin (blue) overlapped.

### Fluorescence microscopy

The conditions and settings for fluorescence were used the same as we described before^8^. Cells grown on coverslips in 35-mm Petri dishes were incubated with a corresponding probe for indicated time in FluoroBright DMEM medium. Subsequently cells were washed and observed alive using a fluorescence microscope DM IRB (Leica) with filter cube I3 (excitation filter BP 450–490 nm and long pass filter LP 515 nm for emission) for green fluorescence. Filter cube N2.1 (excitation filter BP 515–560 nm and long pass filter LP 590 nm for emission) was used for red fluorescence, and filter cube A (excitation filter BP 340–380 nm and long pass filter LP 425 nm for emission) for blue fluorescence. The fluorescence images were acquired by a DFC 480 camera using a 63 × oil immersion objective.

## Supplementary information

Supplementary Information

## Data Availability

All data generated or analyzed during this study are included in this published article and its Supplementary information file.
