# Supplementary material for: Influence of fluorophore and linker length on the localization and trafficking of fluorescent sterol probes
Source: Sci Rep. 2020 Dec 16;10:22053. doi: 10.1038/s41598-020-78085-9 (PMC7745015; doi:10.1038/s41598-020-78085-9)
Supplement: Supplementary file 1 — Supplementary Information [file 41598_2020_78085_MOESM1_ESM.pdf]

Supplementary Information

Influence of fluorophore and linker length on the localization and trafficking of fluorescent sterol probes

Jarmila Králová[1, *], Michal Jurášek[2], Lucie Mikšátková[2], Anna Marešová[2], Jan Fähnrich[2], Petra Cihlářová[2], Pavel Drašar[2], Petr Bartůněk[1] and Vladimír Král[2,†]

[1]CZ-OPENSCREEN, Institute of Molecular Genetics of the Czech Academy of Sciences, v.v.i., Vídeňská 1083, 14220 Prague 4, Czech Republic

[2]University of Chemistry and Technology, Technická 5, 16628 Prague 6, Czech Republic

Table of contents

1. Structures of probes and their building blocks s2
2. Synthesis and structural characterization s3
3. Additional photophysical data s12
4. Cell Imaging s18
5. References s23

1. Structures of probes and their building blocks

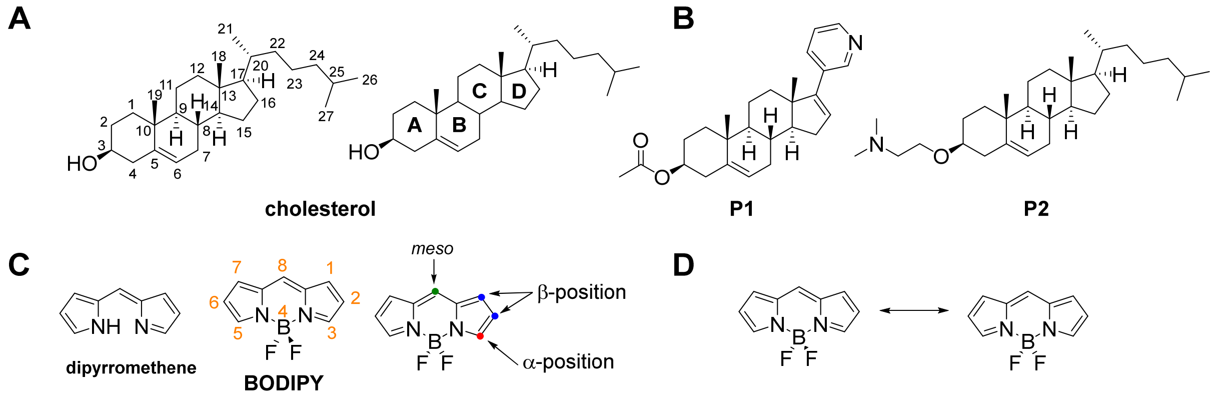

Figure S1. General structure of cholesterol and used building blocks for sterol probe synthesis. (A)The chemical structure of cholesterol with atom numbering. (B) Precursors **P1** and **P2**, (C) BODIPY system and (D) its resonance structures.

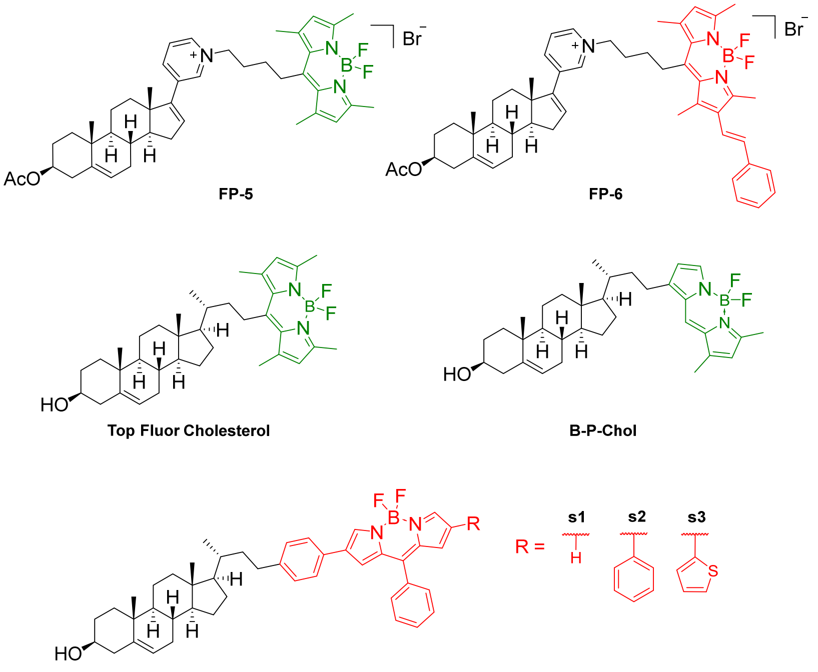

Figure S2. Published fluorescent sterol probes based on steroidal backbone and BODIPY fluorophore: shown are **FP-5** [1], **TopFluor Cholesterol** [2,3], and **B-P-Chol** [4]. Red-shifted **FP-6** probe [1] and cholesterol-BODIPY analogues **s1** – **s3** [5] are depicted.

2. Synthesis and structural characterization

NMR Spectra were recorded on Agilent 400-MR DDR2 (Agilent, Santa Clara, USA). Chemical shifts (δ) are given in ppm and interaction constants (*J*) in Hz. High-resolution mass spectra (HRMS) were measured by LTQ ORBITRAP VELOS with $HESI^+/HESI^-$ ionization (Thermo Scientific). For thin-layer chromatograms were used plates coated with silica gel by Stahl (10-40 µm, Merck) and aluminum TLC sheets-coated silica gel bounded with starch for detection in UV light (Silufol UV 254 nm, Merck, Darmstadt, Germany). For visualization, 50% sulfuric acid in MeOH was used and plates were successively heated. For column chromatography, silica gel (32-62 µm, SiliTech, MP Biomedicals, California, USA) was used. Solvents were purchased from PENTA (Prague, Czech Republic), steroids from Steraloids (Newport, USA) and other reagents from Sigma-Aldrich (Corp., St. Louis, USA).

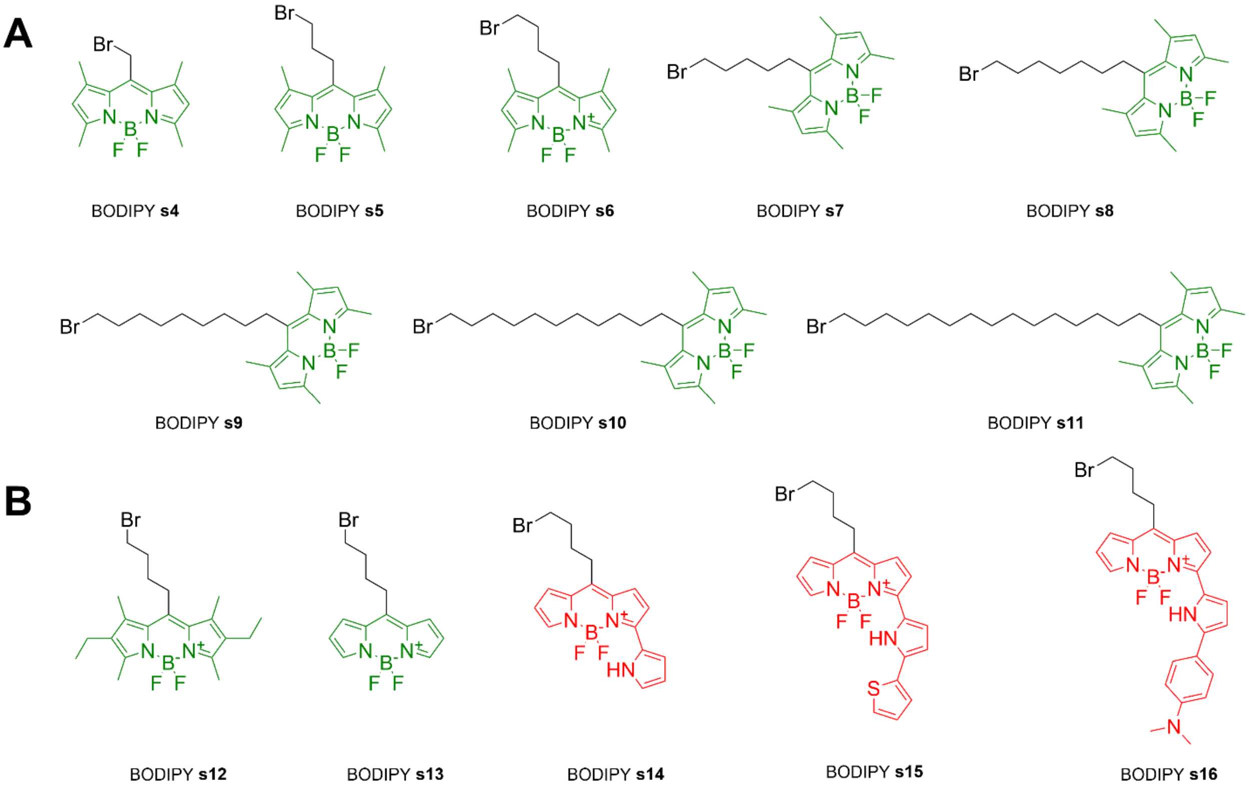

Figure S3. Structures of bromo-BODIPY fluorophores used for the steroid labelling.

General procedure for BODIPYs4-s11 (see Figure S4): To a solution of bromo acid (7.96 mmol) in DCM (50 mL) oxalylchloride (2 mL) and three drops of DMF were added. The mixture was stirred overnight at RT after which the solvents were removed under reduced pressure and the residue co-evaporated with toluene (3 × 10 mL). Product was used without further work-up. To acyl chloride (in case of **s4** preparation bromoacetylbromide was used) in dry DCM (50 mL) 2,4-dimethylpyrrole (1.6 g, 16.72 mmol) was added and the mixture was stirred at RT overnight (in case of **s4** 90 min). Then DCM (50 mL) was added followed by Et_3N (8 mL) and after 20 min $BF_3·Et_2O$ (12 mL). The mixture was stirred additional 3 h at RT. Thereafter, MeOH (40 mL) was added and stirring was continued for 20 min. The solvents were evaporated

under reduced pressure and the residue was chromatographed hexanes-DCM (3/2→1/1, *v/v*, resp.) to obtain product, which was crystallized from MeOH.

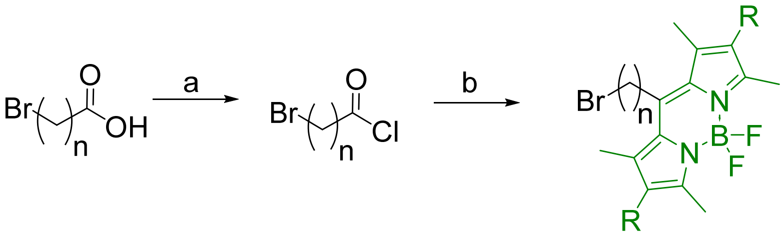

Figure S4. Syntheses of bromo-BODIPY dyes with various linker length. *Reagents and conditions*: a) $(COCl)_2$, DCM, RT, ON, DMF (cat.); b) *i.* 2,4-dimethylpyrrole or 3-ethyl-2,4-dimethylpyrrole, DCM, RT, ON (90 min in case of **s4** synthesis, 0°C→RT); *ii.* Et_3N, 20 min; *iii.* $BF_3 \cdot Et_2O$, 3 h;

BODIPY **s4**: From bromoacetyl bromide (1.6 g, 7.96 mmol) was obtained BODIPY **s4** (0.732 g, 2.14 mmol) as magenta-colored solids in 27% yield. ^1H NMR (400 MHz, $CDCl_3$) δ ppm: 2.54 (br s, 6 H), 2.55 (s, 6 H), 4.68 (s, 2 H), 6.10 (s, 2 H). ^{13}C NMR (101 MHz, $CDCl_3$) δ ppm: 14.67 (t, *J*=2.7 Hz), 15.88, 24.51, 109.99, 122.28, 130.98, 137.24, 140.87, 156.43. HRMS-ESI: *monoisotopic mass* 340.05580 Da, found *m/z* 341.06447 (clcd 341.06333) corresponding to $[M+H]^+$ ion of the proposed structure.

BODIPY **s5**: From bromobutyric acid (1.32 g, 7.96 mmol) was obtained BODIPY **s5** (0.97 g, 2.63 mmol) as orange solids in 33% yield. R_F=0.2 in toluene. ^1H NMR (400 MHz, $CDCl_3$) δ ppm: 2.12 - 2.20 (m, 2 H), 2.44 (s, 6 H), 2.52 (s, 6 H), 3.06 - 3.16 (m, 2 H), 3.55 (t, *J*=6.3 Hz, 2 H), 6.06 (s, 2 H). ^{13}C NMR (101 MHz, $CDCl_3$) δ ppm: 14.48 (t, *J*=2.3 Hz), 16.66, 27.17, 32.90, 33.96, 121.84, 131.42, 140.30, 144.17, 154.37. HRMS-ESI: *monoisotopic mass* 368.08710 Da, found *m/z* 369.09431 (clcd 369.09801) corresponding to $[M+H]^+$ ion of the proposed structure.

BODIPY **s7**: From bromohexanoic acid (1.55 g, 7.96 mmol) was obtained BODIPY **s7** (0.81 g, 2.04 mmol) as orange solids in 26% yield. ^1H NMR (400 MHz, $CDCl_3$) δ ppm 1.66 (dt, *J*=7.6, 3.6 Hz, 4 H), 1.93 (quin, *J*=6.7 Hz, 2 H), 2.42 (s, 6 H), 2.52 (s, 6 H), 2.91 - 3.00 (m, 2 H), 3.44 (t, *J*=6.7 Hz, 2 H), 6.06 (s, 2 H). ^{13}C NMR (101 MHz, $CDCl_3$) δ ppm: 14.45 (t, *J*=2.7 Hz), 16.39, 28.19, 28.56, 30.88, 32.18, 33.37, 121.67, 131.36, 140.23, 145.89, 153.91. HRMS-ESI: *monoisotopic mass* 396.11840 Da, found *m/z* 397.12572 (clcd 397.12600) corresponding to $[M+H]^+$ ion of the proposed structure.

BODIPY **s8**: From bromooctanoic acid (1.78 g, 7.96 mmol) was obtained BODIPY **s8** (1.03 g, 2.42 mmol) as orange solids in 30% yield. ^1H NMR (400 MHz, $CDCl_3$) δ ppm: 1.33 - 1.44 (m, 2 H), 1.45 - 1.56 (m, 4 H), 1.59 - 1.70 (m, 2 H), 1.83 - 1.92 (m, 2 H), 2.42 (s, 6 H), 2.52 (s, 6 H), 2.89 - 2.98 (m, 2 H), 3.39 - 3.45 (m, 2 H), 6.06 (s, 2 H). ^{13}C NMR (101 MHz, $CDCl_3$) δ ppm: 14.43 (t, *J*=2.7 Hz), 16.38, 28.08, 28.37, 28.60, 30.17, 31.79, 32.67, 33.78, 121.57, 131.41, 140.22, 146.40, 153.76. HRMS-ESI: *monoisotopic mass* 424.14970 Da, found *m/z* 425.15678 (clcd 425.15734) corresponding to $[M+H]^+$ ion of the proposed structure.

BODIPY **s9**: From bromodecanoic acid (2 g, 7.96 mmol) was obtained BODIPY **s9** (1.11 g, 2.45 mmol) as orange solids in 30% yield. ^1H NMR (400 MHz, $CDCl_3$) δ ppm: 1.26 - 1.38 (m, 6 H), 1.39 - 1.54 (m, 4 H), 1.56 - 1.69 (m, 2 H), 1.86 (dt, *J*=14.4, 7.1 Hz, 2 H), 2.40 (s, 6 H), 2.52 (s, 6 H), 2.84 - 2.99 (m, 2 H), 3.41 (br t, *J*=6.8 Hz, 2 H), 6.05 (s, 2 H). ^{13}C NMR (101 MHz, $CDCl_3$) δ ppm: 14.42 (t, *J*=2.7 Hz), 16.33, 28.07, 28.42, 28.65, 29.27, 29.33, 30.30, 31.85, 32.73, 33.96, 121.53, 131.41, 140.27, 146.62, 153.65. HRMS-

ESI: *monoisotopic mass* 452.18100 Da, found *m/z* 453.18898 (clcd 453.18867) corresponding to $[M+H]^+$ ion of the proposed structure.

BODIPY **s10**: From bromododecanoic acid (2.22 g, 7.96 mmol) was obtained BODIPY **s10** (720 mg, 1.5 mmol) as orange solids in 19% yield. ^{1}H NMR (400 MHz, $CDCl_3$) δ ppm: 1.22 - 1.37 (m, 10 H), 1.38 - 1.54 (m, 4 H), 1.57 - 1.69 (m, 2 H), 1.80 - 1.92 (m, 2 H), 2.42 (s, 6 H), 2.52 (s, 6 H), 2.89 - 2.98 (m, 2 H), 3.42 (t, *J*=6.8 Hz, 2 H), 6.06 (s, 2 H). ^{13}C NMR (101 MHz, $CDCl_3$) δ ppm: 14.44 (t, *J*=2.7 Hz), 16.37, 28.13, 28.48, 28.72, 29.39, 29.44, 29.49, 30.38, 31.89, 32.80, 34.06, 121.54, 131.41, 140.27, 146.66, 153.67. HRMS-ESI: *monoisotopic mass* 480.21230 Da, found *m/z* 481.22022 (clcd 481.22001) corresponding to $[M+H]^+$ ion of the proposed structure.

BODIPY **s11**: From bromohexadecanoic acid (2.67 g, 7.96 mmol) was obtained BODIPY **s11** (1.33 g, 2.47 mmol) as orange solids in 31% yield. ^{1}H NMR (400 MHz, $CDCl_3$) δ ppm: 1.24 - 1.37 (m, 18 H), 1.38 - 1.53 (m, 6 H), 1.57 - 1.68 (m, 2 H), 1.86 (dt, *J*=14.6, 7.0 Hz, 2 H), 2.42 (s, 6 H), 2.52 (s, 6 H), 2.89 - 2.97 (m, 2 H), 3.42 (t, *J*=6.8 Hz, 2 H), 6.06 (s, 2 H). ^{13}C NMR (101 MHz, $CDCl_3$) δ ppm: 14.43 (t, *J*=2.7 Hz), 16.36, 28.17, 28.49, 28.77, 29.41, 29.44, 29.53, 29.56, 29.57, 29.59, 29.61, 30.40, 31.89, 32.83, 34.09, 121.52, 131.41, 140.28, 146.70, 153.66. HRMS-ESI: *monoisotopic mass* 536.27490 Da, found *m/z* 537.28121 (clcd 537.28272) corresponding to $[M+H]^+$ ion of the proposed structure.

BODIPY **s12**: From 5-bromovaleroyl chloride (1.58 g, 7.96 mmol) was obtained BODIPY **s12** (680 mg, 1.55 mmol) as red crystals in 19 % yield. ^{1}H NMR (400 MHz, $CDCl_3$) δ ppm: 1.06 (t, *J*=7.4 Hz, 6 H), 1.74 - 1.85 (m, 2 H), 2.02 - 2.11 (m, 2 H), 2.34 (s, 6 H), 2.41 (q, *J*=7.6 Hz, 4 H), 2.51 (s, 6 H), 2.96 - 3.04 (m, 2 H), 3.46 (t, *J*=6.5 Hz, 2 H). ^{13}C NMR (101 MHz, $CDCl_3$) δ ppm: 12.42 (t, *J*=3.1 Hz), 13.37, 14.84, 17.18, 27.55, 30.10, 32.83, 33.10, 130.85, 132.70, 135.57, 143.61, 152.33. HRMS-ESI: *monoisotopic mass* 438.16535 Da, found *m/z* 477.12810 (clcd 477.12906) corresponding to $[M+K]^+$ ion of the proposed structure.

BODIPY **s13** and **s14**: To a solution of pyrrole (5.36 g, 80 mmol) in DCE (15 mL) was added dropwise bromovaleroyl chloride (1.58 g, 7.96 mmol) at RT on air. The mixture was stirred 6 h at RT after which TEA (6 mL) followed by $BF_3 \cdot Et_2O$ (10 mL) were added. The mixture was stirred at 50°C on air overnight. The solvents were evaporated under reduced pressure and the residue was chromatographed (hexanes-DCM, 1/1, *v/v*, resp.). The fractions containing both products were re-chromatographed once in the same mobile system.

BODIPY **s13** (299 mg, 0.91 mmol) was obtained as brick-colored solids in 11% yield. R_F = 0.3 in hexanes-AcOEt, 5/1 (*v/v*, resp.; green spot). ^{1}H NMR (400 MHz, $CDCl_3$) δ ppm: 1.91 - 2.05 (m, 4 H), 2.94 (t, *J*=7.4 Hz, 2 H), 3.44 (t, *J*=6.1 Hz, 2 H), 6.55 (d, *J*=3.5 Hz, 2 H), 7.29 (d, *J*=4.3 Hz, 2 H), 7.86 (s, 2 H). ^{13}C NMR (101 MHz, $CDCl_3$) δ ppm: 30.0, 31.8, 32.3, 32.7, 118.2, 118.2, 127.8, 135.1, 143.6, 149.9. HRMS-ESI: *monoisotopic mass* 326.04015 Da, found *m/z* 327.04683 (clcd 327.04797) corresponding to $[M+H]^+$ ion of the proposed structure.

BODIPY **s14** (265 mg, 0.68 mmol) was obtained as purple solids in 9 % yield. R_F = 0.45 in hexanes-AcOEt, 5/1 (*v/v*, resp., pink spot). ^{1}H NMR (400 MHz, $CDCl_3$) δ ppm: 1.86 - 2.04 (m, 4 H), 2.86 (t, *J*=7.4 Hz, 2 H), 3.44 (t, *J*=6.1 Hz, 2 H), 6.37 - 6.41 (m, 1 H), 6.48 (dd, *J*=3.9, 2.3 Hz, 1 H), 6.91 (d, *J*=4.7 Hz, 1 H), 6.99 - 7.05 (m, 2 H), 7.19 (td, *J*=2.9, 1.2 Hz, 1 H), 7.31 (d, *J*=4.7 Hz, 1 H), 7.62 (s, 1 H), 10.50 (br s, 1 H). ^{13}C NMR (101 MHz, $CDCl_3$) δ ppm: 29.47, 31.34, 32.38, 32.95, 111.55, 115.68, 118.34, 120.74, 121.61, 123.46, 126.16, 129.73, 133.24, 136.28, 137.64, 140.93, 151.29. HRMS-ESI: *monoisotopic mass* 391.06670 Da, found *m/z* 392.07330 (clcd 392.07428) corresponding to $[M+H]^+$ ion of the proposed structure.

BODIPY **s15** and **s16**: (*i*) To a solution of BODIPY **s14** (1 g, 2.55 mmol) in THF (35 mL) cooled to 0°C (ice-bad), NBS (454 mg, 2.55 mmol) in THF was added *via* Pasteur pipette dropwise. The mixture was stirred for 30 min. The solvents were removed under reduced pressure and the residue was chromatographed

(hexanes-DCM, 3/2, *v/v*, resp.). The material thus obtained was precipitated with hexanes and the solids were collected by filtration and dried *in vacuo*. BODIPY **s5** (780 mg, 1.66 mmol) was isolated as a dark solid in 65 % yield. R_F = 0.55 in hexanes-AcOEt, 5/1 (*v/v*, resp.). ^{1}H NMR (400 MHz, $CDCl_3$) δ ppm: 1.88 - 2.04 (m, 4 H), 2.85 - 2.90 (m, 2 H), 3.45 (t, *J*=6.1 Hz, 2 H), 6.34 (dd, *J*=3.9, 2.3 Hz, 1 H), 6.50 (dd, *J*=3.9, 2.0 Hz, 1 H), 6.85 (d, *J*=4.7 Hz, 1 H), 6.89 (dd, *J*=3.9, 2.7 Hz, 1 H), 7.05 (d, *J*=3.9 Hz, 1 H), 7.31 (d, *J*=5.1 Hz, 1 H), 7.66 (s, 1 H), 10.44 (br t, *J*=9.8 Hz, 1 H). ^{13}C NMR (101 MHz, $CDCl_3$) δ ppm: 29.57, 31.40, 32.36, 32.88, 108.04, 113.80, 116.14, 118.82, 119.85, 122.44, 124.67, 129.59, 133.49, 137.25, 137.45, 141.85, 149.50. HRMS-ESI: *monoisotopic mass* 468.97721 Da, found *m/z* 469.98400 (clcd 469.98503) corresponding to $[M+H]^+$ ion of the proposed structure.
(ii) To a solution of bromide (above – procedure i.) (200 mg, 0.42 mmol) and 2-thienylboronic acid (350 mg, 2.12 mmol) or 4-(dimethylamino)phenylboronic acid (350 mg, 2.12 mmol) in AcCN (6-10 mL), aqueous Na_2CO_3 (449 mg, 4.24 mmol, in 2 mL) was added and the mixture was sonicated for 5 minutes. Then $Pd(dppf)_2Cl_2$ (69 mg, 20 mol%) was added and the mixture was stirred for 1 hours at 80°C. The heating was removed and the mixture was poured into AcOEt (100 mL) and washed with brine (2×100 mL). The organic layer was dried over Na_2SO_4, filtered and the solvents were evaporated under reduced pressure. The residue was chromatographed.

BODIPY **s15**: Chromatography in hexanes-DCM, 1/1 (*v/v*, resp.). The material obtained was dissolved in AcOEt and precipitated by the addition of hexanes. The solids were collected by filtration and washed with hexanes. Product **s15** (120 mg, 0.25 mmol) was obtained as dark blue solid in 60 % yield. R_F=0.4 hexanes-DCM, 3/2 (*v/v*, resp; blue spot). ^{1}H NMR (400 MHz, $CDCl_3$) δ ppm: 1.88 - 2.02 (m, 4 H), 2.86 (t, *J*=7.4 Hz, 2 H), 3.44 (t, *J*=6.3 Hz, 2 H), 6.48 (dd, *J*=3.9, 2.0 Hz, 1 H), 6.57 (dd, *J*=3.9, 2.3 Hz, 1 H), 6.91 (d, *J*=4.7 Hz, 1 H), 6.99 (d, *J*=3.9 Hz, 1 H), 7.01 (dd, *J*=4.1, 2.5 Hz, 1 H), 7.10 (dd, *J*=5.1, 3.5 Hz, 1 H), 7.26 - 7.31 (m, 2 H), 7.34 (dd, *J*=3.7, 1.0 Hz, 1 H), 7.64 (s, 1 H), 10.72 (br t, *J*=8.4 Hz, 1 H). ^{13}C NMR (101 MHz, $CDCl_3$) δ ppm: 29.48, 31.31, 32.41, 32.96, 110.00, 115.61, 120.13, 120.89, 121.23, 123.34, 123.89, 125.16, 128.20, 129.40, 133.44, 134.47, 134.50, 136.05, 137.99, 139.84, 150.07. HRMS-ESI: *monoisotopic mass* 473.05442 Da, found *m/z* 474.06210 (clcd 474.06224) corresponding to $[M+H]^+$ ion of the proposed structure.

BODIPY **s16** (152 mg, 0.30 mmol) was obtained as dark blue solids in 56 % yield. R_F=0.15 hexanes-DCM, 1/1 (*v/v*, resp.). ^{1}H NMR (400 MHz, $CDCl_3$) δ ppm: 1.86 - 2.03 (m, 4 H), 2.84 (br t, *J*=7.2 Hz, 2 H), 3.03 (s, 6 H), 3.44 (t, *J*=6.1 Hz, 2 H), 6.47 (dd, *J*=3.7, 2.2 Hz, 1 H), 6.60 (dd, *J*=3.7, 2.2 Hz, 1 H), 6.79 (d, *J*=8.6 Hz, 2 H), 6.90 - 6.94 (m, 2 H), 7.09 (dd, *J*=4.3, 2.3 Hz, 1 H), 7.26 (d, *J*=5.1 Hz, 1 H), 7.59 (d, *J*=9.0 Hz, 2 H), 7.61 (br s, 1 H), 10.80 (br t, *J*=8.8 Hz, 1 H). ^{13}C NMR (101 MHz, $CDCl_3$) δ ppm: 29.33, 31.12, 32.45, 33.09, 40.30, 108.44, 112.49, 114.79, 118.77, 119.37, 121.33, 121.59, 123.21, 126.07, 129.25, 133.24, 134.12, 137.06, 138.33, 142.04, 150.30, 150.46. HRMS-ESI: *monoisotopic mass* 510.14020 Da, found *m/z* 511.14800 (clcd 511.14802) corresponding to $[M+H]^+$ ion of the proposed structure.

P1 based probes FP-15 – 26 (Figure S5): A mixture of **P1** (0.38 mmol) and BODIPY **s1-s7** (0.46 mmol) in dry AcCN (8 mL) was stirred in screw-cap vial at 110°C for 24-48h. Then the heating was removed and the mixture was poured into ether-hexanes mixture (1/1, *v/v*, resp.; 100 mL). Solvents were decanted and the residue passed through a short silicagel column ($CHCl_3$-MeOH, 20/1→10/1, *v/v*, resp.). The product was dissolved in DCM and precipitated into Et_2O-hexane mixture (1/1, *v/v*, resp.; 100 mL). The solids were collected by filtration and lyophilized from 1,4-dioxane. The products were stored in plastic vials in freezer at the dark.

P2 based probes FP-27 – 29 (Figure S5): A solution of **P2** (1 eqviv.) and **BODIPY** dye (1.2 eqviv.) in AcOEt-MeOH (1/1, resp.; 4 mL) was stirred in screw caped reactor at 80°C for 5 days. The solvent was evaporated under reduced pressure and the residue was chromatographed on short silica gel column ($CHCl_3$-MeOH, 10/1, *v/v*, resp.). The product was again precipitated into hexane and the collected product was lyophilized from 1,4-dioxane. The products thus obtained were stored in plastic vials in freezer at the dark.

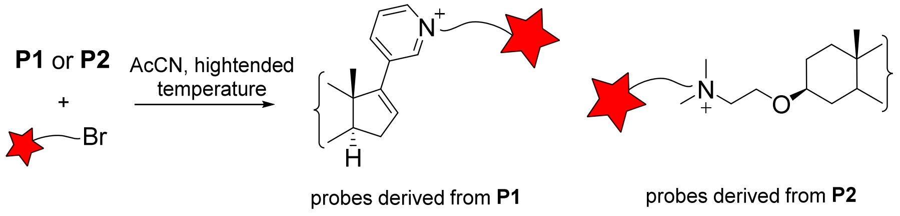

Figure S5. The synthesis of quaternary ammonium steroid-BODIPY salts.

Probe **FP-15**: From **P1** (150 mg, 0.38 mmol) and BODIPY **s4** (156 mg, 0.46 mmol) was obtained **FP-15** (122 mg, 0.167 mmol) as brick colored solids in 44% yield. ^{1}H NMR (400 MHz, $CDCl_3$) δ ppm: 1.07 (s, 6 H), 1.09 - 1.25 (m, 2 H), 1.47 (td, *J*=12.0, 5.3 Hz, 1 H), 1.53 - 1.92 (m, 11 H), 2.04 (s, 3 H), 2.05 - 2.17 (m, 2 H), 2.28 - 2.41 (m, 3 H), 2.46 (s, 6 H), 2.51 (s, 3 H), 2.64 (s, 3 H), 4.54 - 4.66 (m, 1 H), 5.40 (br d, *J*=4.7 Hz, 1 H), 6.21 (s, 1 H), 6.42 (q, *J*=14.1 Hz, 2 H), 6.56 (dd, *J*=3.1, 2.0 Hz, 1 H), 7.16 (s, 1 H), 7.88 (dd, *J*=7.8, 6.3 Hz, 1 H), 8.23 (br d, *J*=8.2 Hz, 1 H), 9.14 (s, 1 H), 9.43 (br d, *J*=5.9 Hz, 1 H). ^{13}C NMR (101 MHz, $CDCl_3$) δ ppm: 14.86 (t, *J*=2.7 Hz), 16.36, 17.00, 17.15, 17.77, 19.21, 20.66, 21.42, 27.67, 30.19, 31.29, 32.27, 34.68, 36.69, 36.85, 38.05, 47.37, 49.95, 56.97, 57.22, 73.71, 122.02, 122.80, 124.02, 127.06, 136.70, 137.27, 138.59, 139.96, 140.24, 140.96, 142.41, 142.79, 144.22, 146.37, 147.46, 160.11, 170.52. HRMS-ESI: *monoisotopic mass* 652.38804 Da (calculated for cation), found *m/z* 652.38955 corresponding to $[M]^+$ ion of the proposed structure.

Probe **FP-16**: From **P1** (150 mg, 0.38 mmol) and BODIPY **s5** (170 mg, 0.46 mmol) was obtained **FP-16** (249 mg, 0.33 mmol) as orange solids in 85% yield. ^{1}H NMR (400 MHz, $CDCl_3$) δ ppm: 1.05 (s, 3 H), 1.06 (s, 3 H), 1.08 - 1.20 (m, 2 H), 1.35 - 1.48 (m, 1 H), 1.50 - 1.79 (m, 7 H), 1.79 - 1.92 (m, 2 H), 2.03 (s, 3 H), 2.04 - 2.14 (m, 2 H), 2.27 (s, 6 H), 2.30 - 2.39 (m, 4 H), 2.44 (s, 6 H), 3.12 - 3.19 (m, 2 H), 4.59 (ddd, *J*=15.8, 10.6, 5.7 Hz, 1 H), 5.26 - 5.47 (m, 3 H), 5.99 (s, 2 H), 6.77 (dd, *J*=2.9, 1.8 Hz, 1 H), 7.85 (dd, *J*=8.2, 5.9 Hz, 1 H), 8.25 (br d, *J*=8.2 Hz, 1 H), 9.47 (br d, *J*=5.9 Hz, 1 H), 9.49 (s, 1 H). ^{13}C NMR (101 MHz, $CDCl_3$) δ ppm: 14.48, 16.47, 16.80, 19.21, 20.68, 21.42, 24.64, 27.64, 30.12, 31.23, 32.34, 33.54, 34.75, 36.68, 36.82, 38.03, 47.31, 49.89, 57.21, 60.52, 73.68, 121.95, 122.14, 127.58, 131.23, 138.05, 138.13, 139.96, 140.43, 140.52, 142.29, 142.35, 142.97, 146.81, 154.65, 170.51. HRMS-ESI: *monoisotopic mass* 680.41934 Da (calculated for cation), found *m/z* 680.42105 corresponding to $[M]^+$ ion of the proposed structure.

Probe **FP-17**: From **P1** (150 mg, 0.38 mmol) and BODIPY **s7** (182 mg, 0.46 mmol) was obtained **FP-17** (228 mg, 0.29 mmol) as brick-colored solids in 75% yield. ^{1}H NMR (400 MHz, $CDCl_3$) δ ppm: 1.07 (s, 6 H), 1.08 - 1.20 (m, 2 H), 1.38 - 1.93 (m, 16 H), 2.03 (s, 3 H), 2.04 - 2.16 (m, 4 H), 2.28 - 2.36 (m, 2 H),

2.38 (s, 6 H), 2.47 (s, 6 H), 2.95 (br t, *J*=7.0 Hz, 2 H), 4.54 - 4.65 (m, 1 H), 4.97 - 5.13 (m, 2 H), 5.40 (br d, *J*=4.7 Hz, 1 H), 6.02 (s, 2 H), 6.75 (dd, *J*=3.1, 1.6 Hz, 1 H), 7.90 (dd, *J*=8.2, 5.9 Hz, 1 H), 8.26 (d, *J*=8.2 Hz, 1 H), 9.30 (s, 1 H), 9.34 (d, *J*=6.3 Hz, 1 H). ^{13}C NMR (101 MHz, $CDCl_3$) δ ppm: 14.45 (t, *J*=2.3 Hz), 16.49, 16.72, 19.21, 20.69, 21.42, 26.32, 27.65, 27.86, 30.15, 31.16, 31.26, 32.09, 32.33, 34.79, 36.69, 36.84, 38.04, 47.35, 49.93, 57.22, 61.43, 73.68, 121.76, 121.95, 127.72, 131.37, 137.75, 138.00, 139.97, 140.49, 141.83, 142.08, 145.70, 146.91, 153.88, 170.51. HRMS-ESI: *monoisotopic mass* 708.45064 Da (calculated for cation), found *m/z* 708.45202 corresponding to $[M]^+$ ion of the proposed structure

Probe **FP-18**: From **P1** (150 mg, 0.38 mmol) and BODIPY **s8** (196 mg, 0.46 mmol) was obtained **FP-18** (238 mg, 0.29 mmol) as orange lyophilizate in 76% yield. ^{1}H NMR (400 MHz, $CDCl_3$) δ ppm: 1.04 (s, 3 H), 1.04 (s, 3 H), 1.06 - 1.17 (m, 2 H), 1.32 - 1.48 (m, 7 H), 1.49 - 1.75 (m, 9 H), 1.77 - 1.91 (m, 2 H), 2.01 (s, 3 H), 2.03 - 2.14 (m, 3 H), 2.27 - 2.33 (m, 3 H), 2.35 (s, 6 H), 2.46 (s, 6 H), 2.82 - 2.92 (m, 2 H), 4.57 (tt, *J*=10.8, 5.3 Hz, 1 H), 4.93 - 5.10 (m, 3 H), 5.37 (br d, *J*=4.7 Hz, 1 H), 6.01 (s, 2 H), 6.71 (dd, *J*=3.1, 2.0 Hz, 1 H), 7.93 (dd, *J*=8.2, 5.9 Hz, 1 H), 8.25 (br d, *J*=8.2 Hz, 1 H), 9.25 (s, 1 H), 9.32 (br d, *J*=5.9 Hz, 1 H). ^{13}C NMR (101 MHz, $CDCl_3$) δ ppm: 14.43, 16.46, 16.49, 19.20, 20.68, 21.41, 26.00, 27.64, 28.21, 28.90, 29.94, 30.13, 31.26, 31.76, 32.20, 32.30, 34.76, 36.67, 36.83, 38.03, 47.32, 49.93, 57.22, 61.72, 73.68, 121.62, 121.95, 127.80, 131.35, 137.57, 137.89, 139.97, 140.37, 140.46, 141.82, 142.17, 146.40, 146.94, 153.66, 170.51. HRMS-ESI: *monoisotopic mass* 736.48194 Da (calculated for cation), found *m/z* 759.46687 (clcd 759.47249) corresponding to $[M+Na]^+$ ion of the proposed structure.

Probe **FP-19**: From **P1** (150 mg, 0.38 mmol) and BODIPY **s9** (208 mg, 0.46 mmol) was obtained **FP-19** (255 mg, 0.30 mmol) as orange lyophilizate in 79% yield. ^{1}H NMR (400 MHz, $CDCl_3$) δ ppm: 1.04 (br s, 3 H), 1.05 (br s, 3 H), 1.09 - 1.15 (m, 1 H), 1.17 - 1.76 (m, 21 H), 1.77 - 1.90 (m, 2 H), 2.01 (s, 3 H), 2.04 - 2.15 (m, 2 H), 2.23 - 2.34 (m, 2 H), 2.37 (s, 6 H), 2.47 (s, 6 H), 2.80 - 2.99 (m, 2 H), 4.58 (ddd, *J*=15.7, 10.4, 5.3 Hz, 1 H), 4.94 - 5.14 (m, 3 H), 5.38 (br d, *J*=3.5 Hz, 1 H), 6.02 (s, 2 H), 6.73 (br s, 1 H), 7.99 (br dd, *J*=7.8, 6.3 Hz, 1 H), 8.27 (br d, *J*=7.8 Hz, 1 H), 9.24 (s, 1 H), 9.35 (br d, *J*=5.9 Hz, 1 H). ^{13}C NMR (101 MHz, $CDCl_3$) δ ppm: 14.42, 16.43, 16.49, 19.19, 20.67, 21.40, 25.24, 25.96, 27.64, 28.36, 28.97, 29.19, 29.24, 30.13, 30.19, 31.26, 31.86, 32.17, 32.30, 34.78, 36.67, 36.83, 38.02, 47.34, 49.93, 57.22, 61.90, 73.67, 121.57, 121.95, 127.86, 131.36, 137.56, 137.83, 139.96, 140.34, 140.49, 141.78, 142.28, 146.59, 146.95, 153.60, 170.50. HRMS-ESI: *monoisotopic mass* 764.51324 Da (calculated for cation), found *m/z* 764.51541 corresponding to $[M]^+$ ion of the proposed structure.

Probe **FP-20**: From **P1** (150 mg, 0.383mmol) and BODIPY **s10** (221 mg, 0.46 mmol) was obtained product (227 mg, 0.26 mmol) as orange solids in 68% yield. ^{1}H NMR (400 MHz, $CDCl_3$) δ ppm: 1.00 (br s, 3 H), 1.02 (br s, 3 H), 1.17 (s, 6 H), 1.22 - 1.35 (m, 5 H), 1.35 - 1.44 (m, 2 H), 1.47 - 1.72 (m, 8 H), 1.73 - 1.86 (m, 2 H), 1.97 (s, 3 H), 2.01 - 2.15 (m, 2 H), 2.19 - 2.29 (m, 2 H), 2.33 (br s, 6 H), 2.43 (br s, 6 H), 2.78 - 2.88 (m, 2 H), 4.45 - 4.58 (m, 1 H), 4.91 - 5.10 (m, 2 H), 5.33 (br s, 1 H), 5.98 (s, 2 H), 6.71 (br s, 1 H), 7.99 (dd, *J*=8.2, 6.3 Hz, 1 H), 8.25 (br d, *J*=8.2 Hz, 1 H), 9.25 (br s, 1 H), 9.31 (br s, 1 H). ^{13}C NMR (101 MHz, $CDCl_3$) δ ppm: 14.39 (t, *J*=2.7 Hz), 16.36, 16.46, 19.16, 20.65, 21.39, 26.00, 27.61, 28.39, 29.03, 29.28, 29.31, 29.36, 29.40, 30.11, 30.28, 31.23, 31.84, 32.20, 32.26, 34.74, 36.64, 36.80, 38.00, 47.29, 49.91, 57.18, 61.88, 73.65, 121.52, 121.92, 127.93, 131.34, 137.47, 137.76, 139.93, 140.34, 140.47, 141.85, 142.22, 146.67, 146.94, 153.51, 170.46. HRMS-ESI: *monoisotopic mass* 792.54454 Da (calculated for cation), found *m/z* 792.54680 corresponding to $[M]^+$ ion of the proposed structure.

Probe **FP-21**: From **P1** (150 mg, 0.38 mmol) and BODIPY **s11** (247 mg, 0.46 mmol) was obtained **FP-21** (266 mg, 0.29 mmol) as orange colored lyophilizate in 74% yield. ^{1}H NMR (400 MHz, $CDCl_3$) δ ppm: 1.05 (br s, 3 H), 1.07 (br s, 3 H), 1.14 - 1.38 (m, 18 H), 1.39 - 1.51 (m, 3 H), 1.53 - 1.78 (m, 7 H), 1.78 - 1.91 (m, 2 H), 2.01 (s, 3 H), 2.04 - 2.15 (m, 2 H), 2.23 - 2.35 (m, 2 H), 2.38 (s, 6 H), 2.48 (s, 6 H), 2.85 - 2.95 (m, 2 H), 4.51 - 4.66 (m, 1 H), 4.96 - 5.16 (m, 2 H), 5.38 (br s, 1 H), 6.02 (s, 2 H), 6.73 (br s, 1 H), 8.01 (br dd, *J*=8.0, 6.1 Hz, 1 H), 8.28 (br d, *J*=8.2 Hz, 1 H), 9.21 (br s, 1 H), 9.37 (br d, *J*=5.9 Hz, 1 H). ^{13}C NMR

(101 MHz, $CDCl_3$) δ ppm: 14.41 (t, *J*=2.7 Hz), 16.36, 16.51, 19.19, 20.67, 21.40, 26.05, 27.64, 28.46, 29.10, 29.35, 29.38, 29.49, 29.51, 29.53, 29.56, 29.58, 30.15, 30.35, 31.26, 31.88, 32.20, 32.30, 34.80, 36.68, 36.83, 38.03, 47.35, 49.94, 57.22, 62.00, 73.67, 121.51, 121.96, 127.87, 131.38, 137.49, 137.79, 139.96, 140.32, 140.53, 141.76, 142.36, 146.72, 146.99, 153.57, 170.48. HRMS-ESI: *monoisotopic mass* 848.60714 Da (calculated for cation), found *m/z* 848.60953 corresponding to $[M]^+$ ion of the proposed structure.

Probe **FP-22**: From **P1** (150 mg, 0.38 mmol) and BODIPY **s13** (150 mg, 0.46 mmol) was obtained **FP-22** (153 mg, 0.21 mmol) as brick colored lyophilizate in 55% yield. ^1H NMR (400 MHz, $CDCl_3$) δ ppm: 1.01 (s, 3 H), 1.04 (s, 3 H), 1.06 - 1.17 (m, 2 H), 1.39 (td, *J*=12.0, 4.5 Hz, 1 H), 1.46 - 1.75 (m, 7 H), 1.77 - 1.91 (m, 4 H), 1.98 - 2.00 (m, 2 H), 2.01 (s, 3 H), 2.04 - 2.22 (m, 4 H), 2.29 - 2.32 (m, 3 H), 3.04 (br t, *J*=7.6 Hz, 2 H), 4.57 (ddd, *J*=15.7, 10.5, 5.5 Hz, 1 H), 4.88 - 5.02 (m, 2 H), 5.37 (br d, *J*=3.9 Hz, 1 H), 6.46 (dd, *J*=3.9, 1.6 Hz, 2 H), 6.61 (br s, 1 H), 7.44 (br d, *J*=3.9 Hz, 2 H), 7.75 (s, 2 H), 7.78 (br dd, *J*=7.8, 6.7 Hz, 1 H), 8.18 (br d, *J*=8.2 Hz, 1 H), 9.09 - 9.18 (m, 2 H). ^{13}C NMR (101 MHz, $CDCl_3$) δ ppm: 16.39, 19.20, 20.64, 21.42, 27.65, 29.70, 30.06, 30.12, 31.24, 31.91, 32.30, 34.65, 36.67, 36.82, 38.04, 47.29, 49.89, 57.19, 61.02, 73.71, 118.34, 121.95, 127.74, 128.84, 135.02, 137.51, 138.08, 139.98, 140.59, 141.72, 141.84, 143.49, 146.94, 149.80, 170.52. HRMS-ESI: *monoisotopic mass* 637.36511 Da (calculated for cation), found *m/z* 638.37420 (clcd 638.37307) corresponding to $[M+H]^+$ ion of the proposed structure.

Probe **FP-23**: From **P1** (150 mg, 0.38 mmol) and BODIPY **s12** (202 mg, 0.46 mmol) was obtained product (139 mg, 0.167 mmol) as orange lyophilizate in 44% yield. ^1H NMR (400 MHz, $CDCl_3$) δ ppm: 1.01 (t, *J*=7.6 Hz, 6 H), 1.04 (s, 3 H), 1.07 (s, 3 H), 1.08 - 1.22 (m, 2 H), 1.38 - 1.47 (m, 1 H), 1.50 - 1.77 (m, 9 H), 1.79 (s, 2 H), 1.81 - 1.94 (m, 2 H), 2.00 - 2.02 (m, 1 H), 2.04 (s, 3 H), 2.06 - 2.25 (m, 4 H), 2.29 (s, 6 H), 2.31 - 2.40 (m, 8 H), 2.47 (s, 6 H), 2.97 - 3.10 (m, 2 H), 4.61 (ddd, *J*=16.0, 10.6, 5.5 Hz, 1 H), 5.05 - 5.21 (m, 2 H), 5.40 (br d, *J*=5.1 Hz, 1 H), 6.72 (dd, *J*=3.1, 1.6 Hz, 1 H), 7.71 (dd, *J*=8.2, 6.3 Hz, 1 H), 8.22 (br d, *J*=8.2 Hz, 1 H), 9.33 - 9.38 (m, 2 H). ^{13}C NMR (101 MHz, $CDCl_3$) δ ppm: 12.44 (t, *J*=3.1 Hz), 13.82, 14.89, 16.43, 17.15, 19.20, 20.66, 21.42, 27.36, 27.67, 27.88, 30.15, 31.27, 32.14, 32.35, 34.74, 36.70, 36.84, 38.05, 47.34, 49.95, 57.19, 60.95, 73.71, 121.99, 127.46, 130.78, 132.92, 136.06, 137.78, 138.01, 139.98, 140.51, 141.98, 142.10, 143.14, 146.87, 152.44, 170.53. HRMS-ESI: *monoisotopic mass* 750.49759 Da (calculated for cation), found *m/z* 789.47388 (calculated 789.46210) corresponding to $[M+K]^+$ ion of the proposed structure.

Probe **FP-24**: From **P1** (150 mg, 0.38 mmol) and BODIPY **s14** (180 mg, 0.46 mmol) was obtained product (76 mg, 0.097 mmol) as magenta colored lyophilizate in 25% yield. ^1H NMR (400 MHz, $CDCl_3$) δ ppm: 0.94 (s, 3 H), 1.00 (s, 3 H), 1.02 - 1.13 (m, 2 H), 1.24 - 1.71 (m, 9 H), 1.72 - 1.86 (m, 4 H), 1.87 - 1.99 (m, 3 H), 2.02 (s, 4 H), 2.07 - 2.40 (m, 7 H), 2.95 (br t, *J*=7.2 Hz, 2 H), 4.57 (tt, *J*=10.6, 5.4 Hz, 1 H), 5.01 (br s, 2 H), 5.35 (br d, *J*=3.9 Hz, 1 H), 6.30 - 6.33 (m, 1 H), 6.35 (br dd, *J*=3.5, 2.0 Hz, 1 H), 6.58 (br s, 1 H), 6.93 (br d, *J*=4.7 Hz, 1 H), 7.03 (br s, 1 H), 7.04 (br s, 1 H), 7.13 (br s, 1 H), 7.46 - 7.50 (m, 1 H), 7.52 (br d, *J*=4.7 Hz, 1 H), 7.62 - 7.68 (m, 1 H), 8.08 (br d, *J*=8.2 Hz, 1 H), 9.15 (br s, 2 H), 10.58 (br t, *J*=9.2 Hz, 1 H). ^{13}C NMR (101 MHz, $CDCl_3$) δ ppm: 16.31, 19.17, 20.60, 21.42, 27.67, 29.22, 29.60, 30.07, 31.20, 31.88, 32.22, 34.62, 36.63, 36.78, 38.04, 47.19, 49.83, 57.06, 60.84, 73.74, 111.80, 115.75, 118.68, 120.99, 121.97, 122.10, 123.24, 126.25, 127.53, 130.96, 133.05, 135.97, 137.46, 137.63, 137.81, 139.94, 140.41, 140.62, 141.63, 141.71, 146.79, 151.32, 170.51. HRMS-ESI: *monoisotopic mass* 703.39894 Da (calculated for cation), found *m/z* 703.40048 corresponding to $[M]^+$ ion of the proposed structure.

Probe **FP-25**: From **P1** (30 mg, 0.077 mmol) and BODIPY **s6** (36 mg, 0.77 mmol) was obtained product (29 mg, 0.033 mmol) as magenta-colored solids in 43% yield. ^1H NMR (400 MHz, $CDCl_3$) δ ppm: 0.93 (s, 3 H), 1.01 (s, 3 H), 1.24 - 2.03 (m, 19 H), 2.05 (s, 3 H), 2.08 - 2.15 (m, 2 H), 2.17 - 2.38 (m, 4 H), 2.90 (br t, *J*=7.6 Hz, 2 H), 4.58 (tt, *J*=10.8, 5.4 Hz, 1 H), 4.68 (br t, *J*=7.6 Hz, 2 H), 5.36 (br d, *J*=4.7 Hz, 1 H), 6.39 - 6.43 (m, 2 H), 6.53 (dd, *J*=3.7, 2.2 Hz, 1 H), 6.90 (d, *J*=4.7 Hz, 1 H), 7.00 - 7.03 (m, 2 H), 7.09 (dd, *J*=4.9, 3.7 Hz, 1 H), 7.30 (dd, *J*=5.1, 1.2 Hz, 1 H), 7.35 (dd, *J*=3.5, 0.8 Hz, 1 H), 7.43 (d, *J*=4.7 Hz, 1 H), 7.54 (s,

1 H), 7.63 - 7.68 (m, 1 H), 8.11 (br d, *J*=7.8 Hz, 1 H), 8.73 (br s, 1 H), 8.76 (br d, *J*=6.3 Hz, 1 H), 10.67 (br t, *J*=9.6 Hz, 1 H). ^{13}C NMR (101 MHz, $CDCl_3$) δ ppm: 16.24, 19.15, 20.59, 21.44, 27.66, 29.22, 29.31, 29.69, 30.06, 31.16, 31.64, 32.22, 34.66, 36.60, 36.73, 38.05, 47.30, 49.80, 57.00, 61.43, 73.75, 110.20, 115.74, 120.57, 121.25, 121.63, 121.96, 123.45, 123.72, 125.25, 127.64, 128.28, 133.22, 134.32, 134.59, 135.68, 137.10, 138.03, 138.08, 139.40, 139.93, 140.74, 141.24, 146.84, 150.07, 170.51. HRMS-ESI: *monoisotopic mass* 785.38666 Da (calculated for cation), found *m/z* 785.38805 corresponding to $[M]^+$ ion of the proposed structure.

Probe **FP-26**: From **P1** (42 mg, 0.11 mmol) and BODIPY **s13** (55 mg, 0.11 mmol) was obtained product (26 mg, 29 μmol) as magenta-colored solids in 26% yield. ^{1}H NMR (400 MHz, DMSO-d_6) δ ppm: 0.72 - 0.88 (m, 2 H), 0.95 (s, 3 H), 0.97 (s, 3 H), 0.99 - 1.32 (m, 6 H), 1.33 - 1.55 (m, 7 H), 1.56 - 1.79 (m, 7 H), 1.95 (s, 3 H), 1.98 - 2.13 (m, 4 H), 2.17 - 2.33 (m, 4 H), 2.96 (s, 6 H), 4.31 - 4.41 (m, 1 H), 4.65 (br t, *J*=6.7 Hz, 2 H), 5.33 (br d, *J*=3.9 Hz, 1 H), 6.43 (dd, *J*=3.9, 2.3 Hz, 1 H), 6.48 (br s, 1 H), 6.75 (br d, *J*=4.3 Hz, 1 H), 6.79 (br d, *J*=9.0 Hz, 2 H), 7.07 (br d, *J*=3.5 Hz, 1 H), 7.43 (br d, *J*=4.7 Hz, 1 H), 7.51 (br d, *J*=3.9 Hz, 2 H), 7.55 (s, 1 H), 7.60 (br d, *J*=9.0 Hz, 1 H), 7.66 (d, *J*=4.7 Hz, 1 H), 8.05 (br dd, *J*=8.2, 5.9 Hz, 1 H), 8.53 (br d, *J*=8.2 Hz, 1 H), 8.90 - 8.96 (m, 2 H). ^{13}C NMR (101 MHz, DMSO-d_6) δ ppm: 16.12, 19.22, 20.57, 21.49, 27.74, 29.15, 29.66, 30.13, 31.23, 32.03, 33.98, 36.61, 38.11, 47.13, 49.83, 57.29, 61.04, 73.56, 112.75, 115.43, 121.75, 122.18, 126.47, 128.36, 131.31, 134.96, 135.71, 136.57, 138.38, 140.30, 141.65, 142.32, 142.99, 147.98, 150.72, 170.15. HRMS-ESI: *monoisotopic mass* 822.47244 Da (calculated for cation), found *m/z* 822.47485 corresponding to $[M]^+$ ion of the proposed structure.

Probe **FP-27**: From **P2** (150 mg, 0.33 mmol) and BODIPY **s1** (149 mg, 0.39 mmol) was obtained product (213 mg, 0.25 mmol) as orange lyophilizate in 77% yield. ^{1}H NMR (400 MHz, $CDCl_3$) δ ppm: 0.85 (d, *J*=2.0 Hz, 3 H), 0.87 (d, *J*=2.0 Hz, 3 H), 0.91 (d, *J*=6.3 Hz, 4 H), 0.96 (s, 3 H), 0.97 - 1.73 (m, 26 H), 1.76 - 1.88 (m, 4 H), 1.89 - 2.15 (m, 7 H), 2.23 - 2.34 (m, 1 H), 2.42 (s, 6 H), 2.48 (s, 6 H), 2.98 - 3.08 (m, 2 H), 3.10 - 3.20 (m, 2 H), 3.33 (s, 6 H), 3.64 - 3.74 (m, 4 H), 3.83 (br dd, *J*=17.6, 4.7 Hz, 4 H), 5.30 - 5.36 (m, 1 H), 6.04 (s, 2 H). ^{13}C NMR (101 MHz, $CDCl_3$) δ ppm: 11.84, 14.48, 16.85, 18.70, 19.31, 21.02, 22.55, 22.81, 22.97, 23.81, 24.25, 27.50, 27.99, 28.17, 28.20, 31.79, 31.86, 35.76, 36.16, 36.72, 36.84, 38.75, 39.49, 39.68, 42.28, 50.01, 51.77, 56.10, 56.65, 61.85, 63.09, 65.30, 80.01, 122.00, 122.59, 131.32, 139.55, 140.53, 144.62, 154.26. HRMS-ESI: *monoisotopic mass* 760.61278 Da (calculated for cation), found *m/z* 760.61662 corresponding to $[M]^+$ ion of the proposed structure.

Probe **FP-28**: From **P2** (150 mg, 0.33 mmol) and BODIPY **s12** (171 mg, 0.39 mmol) was obtained product (185 mg, 0.21 mmol) as dark orange lyophilizate in 62% yield. ^{1}H NMR (400 MHz, $CDCl_3$) δ ppm: 0.66 (s, 3 H), 0.84 (d, *J*=1.6 Hz, 3 H), 0.86 (d, *J*=2.0 Hz, 3 H), 0.90 (d, *J*=6.7 Hz, 3 H), 0.94 (s, 3 H), 1.02 (br t, *J*=7.6 Hz, 6 H), 1.05 - 1.18 (m, 7 H), 1.19 - 1.74 (m, 17 H), 1.76 - 1.88 (m, 4 H), 1.89 - 2.02 (m, 5 H), 2.03 - 2.15 (m, 2 H), 2.24 - 2.31 (m, 1 H), 2.34 (s, 6 H), 2.35 - 2.41 (m, 4 H), 2.47 (s, 6 H), 3.05 - 3.23 (m, 4 H), 3.36 (s, 6 H), 3.61 - 3.71 (m, 2 H), 3.85 (s, 4 H), 5.31 (br d, *J*=5.1 Hz, 1 H). ^{13}C NMR (101 MHz, $CDCl_3$) δ ppm: 11.83, 12.45 (t, *J*=2.7 Hz, 13.87, 14.85, 17.14, 18.69, 19.28, 21.01, 22.54, 22.80, 22.95, 23.81, 24.25, 27.55, 27.99, 28.08, 28.19, 31.79, 31.85, 36.15, 36.70, 36.83, 38.77, 39.48, 39.67, 42.27, 50.00, 51.82, 56.09, 56.65, 61.90, 62.96, 65.25, 80.00, 122.60, 130.82, 132.98, 135.72, 139.51, 142.88, 152.58. HRMS-ESI: *monoisotopic mass* 816.67538 Da (calculated for cation), found *m/z* 816.67882 corresponding to $[M]^+$ ion of the proposed structure.

Probe **FP-29**: From **P2** (150 mg, 0.33mmol) and BODIPY **s14** (152 mg, 0.39 mmol) was obtained product (45 mg, 0.053 mmol) as orange solids in 16 % yield. ^{1}H NMR (400 MHz, $CDCl_3$) δ ppm: 0.65 (s, 3 H), 0.85 (d, *J*=1.6 Hz, 3 H), 0.86 (d, *J*=1.6 Hz, 3 H), 0.89 (br s, 3 H), 0.91 (s, 6 H), 0.94 - 1.17 (m, 9 H), 1.17 - 1.60 (m, 13 H), 1.61 - 2.12 (m, 11 H), 2.22 (br dd, *J*=12.7, 2.5 Hz, 1 H), 2.87 - 2.98 (m, 2 H), 3.00 - 3.13 (m, 1 H), 3.19 (br s, 6 H), 3.46 - 3.57 (m, 1 H), 3.61 (br s, 2 H), 3.68 - 3.81 (m, 2 H), 5.29 (s, 1 H), 6.28 - 6.45 (m, 2 H), 6.96 (br d, *J*=4.3 Hz, 1 H), 7.01 - 7.08 (m, 2 H), 7.15 (br s, 1 H), 7.44 - 7.53 (m, 1 H), 7.54 (s, 1 H), 10.57 (br t, *J*=9.2 Hz, 1 H). ^{13}C NMR (101 MHz, $CDCl_3$) δ ppm: 11.84, 18.71, 19.29, 21.01, 22.55, 22.67, 22.82, 23.84, 24.26, 28.00, 28.05, 28.21, 29.37, 29.67, 31.78, 31.86, 35.78, 36.16, 36.69, 36.78,

38.67, 39.49, 39.68, 42.27, 49.97, 51.80, 56.12, 56.65, 61.62, 63.40, 65.39, 79.87, 111.78, 115.79, 118.74, 121.17, 121.94, 122.43, 123.34, 126.31, 130.87, 133.08, 136.10, 137.74, 139.64, 140.37, 151.49. HRMS-ESI: *monoisotopic mass* 769.56772 Da (calculated for cation), found *m/z* 769.57838 corresponding to $[M+K]^+$ ion of the proposed structure.

Table S1. Calculated lipophilicity of synthetic probes **FP-15 − FP-26** in comparison to cholesterol, cholesteryl acetate and synthetic commercial probes **TF-Cholesterol** and **B-P-Cholesterol**

Probe	Mol. Weight*	ACD/LogP GALAS**	ACD/LogP GALAS RI***	Consensus LogP****
**Cholesterol**	386.65	6.33	0.5604	8.10
**Cholesteryl acetate**	428.69	6.99	0.4522	9.17
**TF-Cholesterol**	576.61	6.31	0.1652	6.31
**B-P-Chol**	548.56	5.97	0.1903	5.97
**FP-5**	694.72	3.02	0.2728	3.02
**FP-15**	652.64	2.49	0.2881	2.49
**FP-16**	680.70	2.83	0.2792	2.83
**FP-17**	708.75	3.46	0.3001	3.46
**FP-18**	736.80	3.98	0.3168	3.98
**FP-19**	764.86	4.54	0.3238	4.54
**FP-20**	792.91	5.13	0.3396	5.13
**FP-21**	849.02	6.19	0.3509	6.19
**FP-22**	638.62	2.23	0.2041	2.23
**FP-23**	750.83	4.26	0.3131	4.26
**FP-24**	703.69	2.76	0.2445	2.76
**FP-25**	785.82	3.45	0.3091	3.45
**FP-26**	822.85	3.81	0.3302	3.81
**FP-27**	760.95	4.91	0.2495	4.91
**FP-28**	817.06	5.81	0.1742	5.81
**FP-29**	769.92	4.45	0.2323	4.45

*LogP are calculated for parent steroidal cation, not for the salt, if applicable. Software name and version: ACD/Percepta 2018.1.1 (Build 3044)

** GALAS (Global, AdjustedLocally According to Similarity) modeling methodology[6]

*** Reliability Index

****The consensus LogP model predicts LogP as a weighted average of ACD/LogP Classic and ACD/LogP GALAS predictions.

3. Additional photophysical data

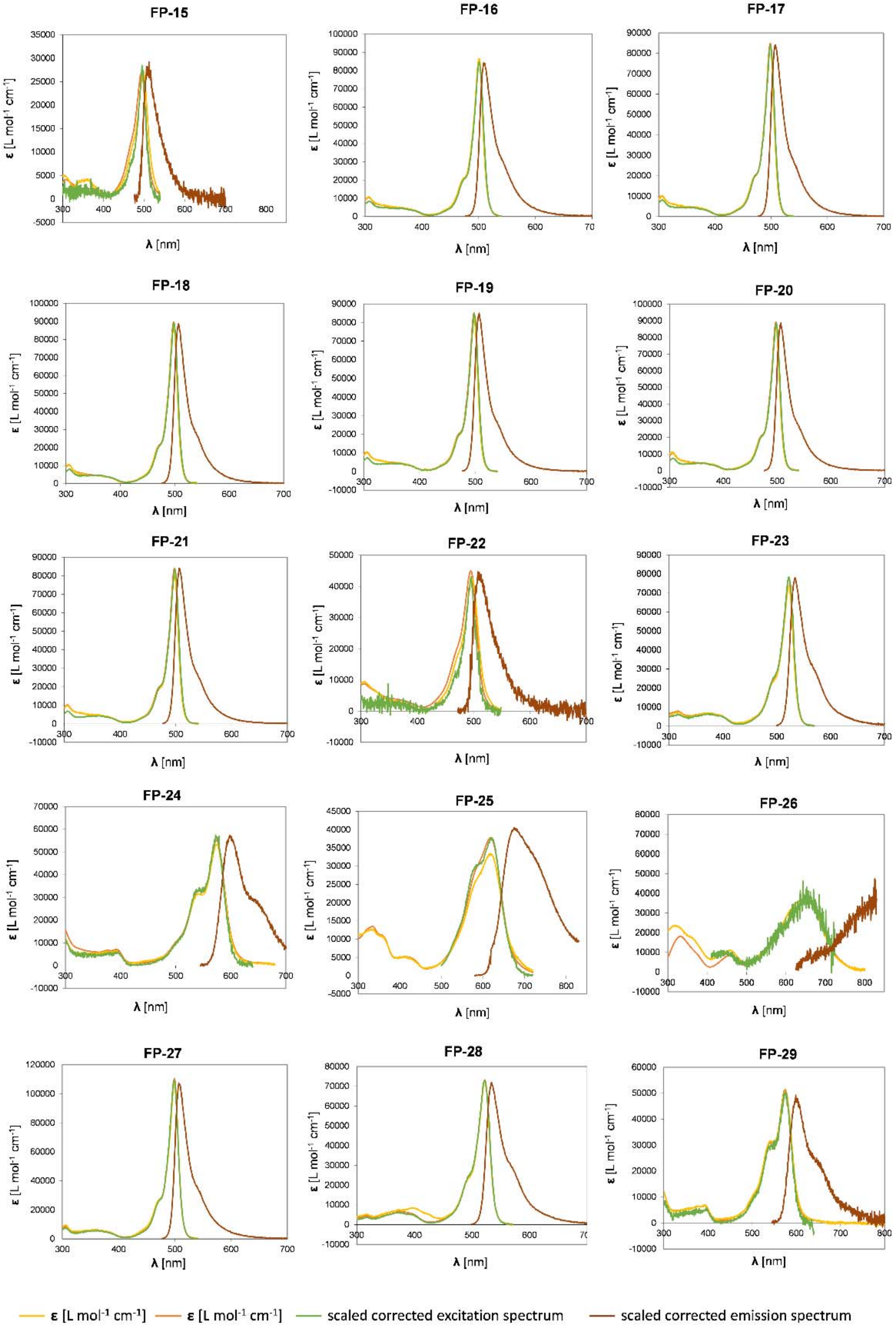

Figure S6: Absorption and emission spectra of fluorescent probes

Table S2. Absorption and fluorescence characteristics of studied compounds.

Compound	λ_{Amax} [nm]	ε [L mol^{-1}cm^{-1}]	λ_{Fmax} [nm]	λ_{Ex} [nm]	λ_{Em} [nm]
**FP-15**	501	85800	510	498, 472	506, 545
**FP-16**	498	84900	507	498, 472	506, 545
**FP-17**	498	85000	507	498, 472	506, 545
**FP-18**	498	89200	507	498, 472	506, 545
**FP-19**	498	89700	507	498, 472	506, 545
**FP-20**	498	84100	507	498, 472	506, 545
**FP-21**	522	77400	534	522, 494	534, 575
**FP-22**	645	37100	>830	645, 608	790, 740
**FP-23**	495[a]	45000	510	494, 467, 470	507, 540
**FP-24**	574	55100	599	574, 540	598, 645
**FP-25**	618	37800	676	620, 575, 580	665, 714
**FP-26**	522	73300	534	522, 494	534, 575
**FP-27**	499	110000	508	499, 472	508, 545
**FP-28**	575	51300	600	574, 540	598, 640
**FP-29**	493[a]	26900	511	494, 472	508, 545

λ_{Amax}, ε - wavelength and absorption coefficient at absorption maximum, λ_{Fmax} - wavelength of maximum of corrected emission spectra, λ_{Ex} , λ_{Em} - wavelengths at which emission and excitation spectra were recorded.

[a] Absorption spectrum shifts by several nm to the red when traces of water are present in DMSO.

FP + cholesterol

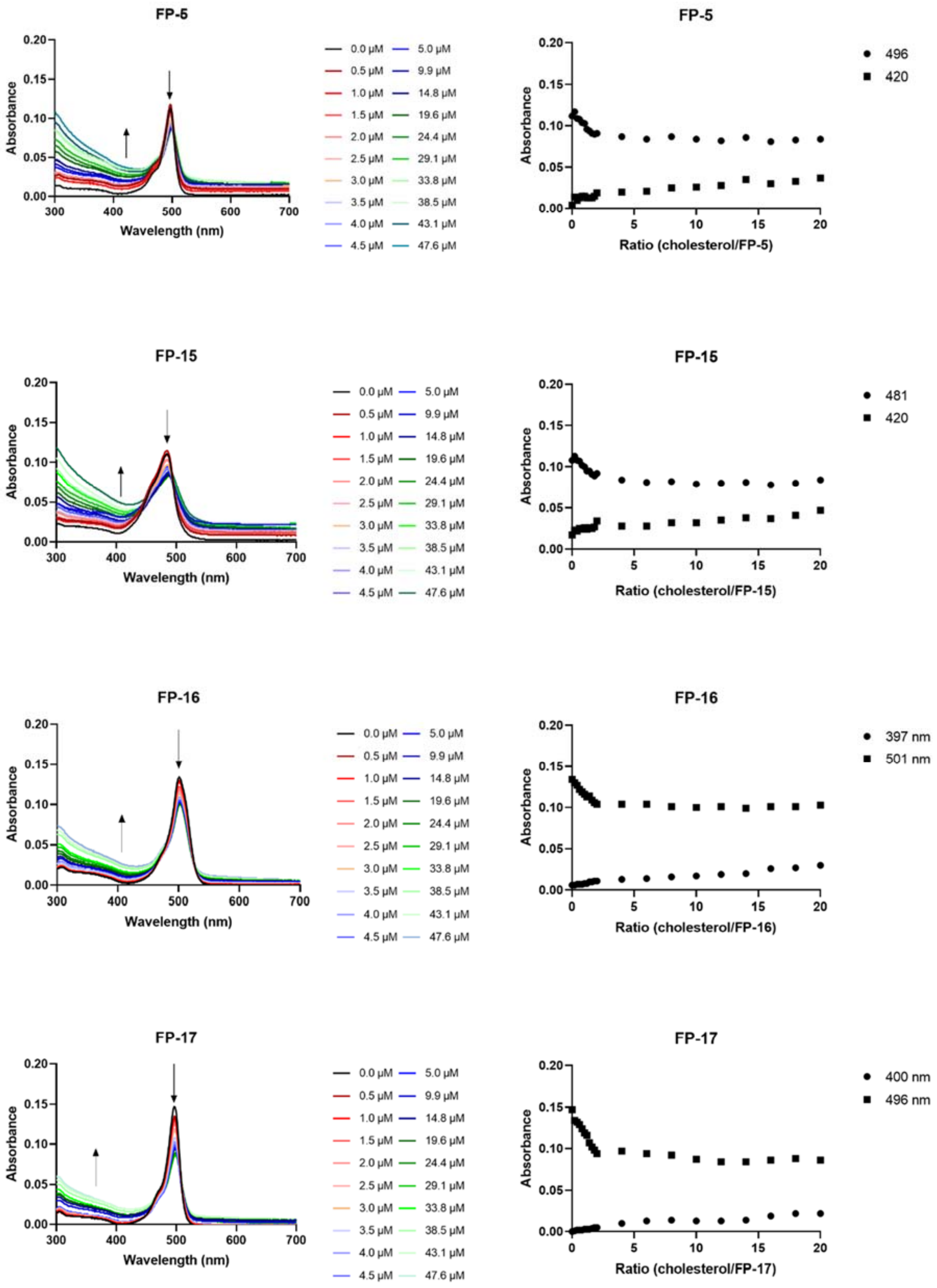

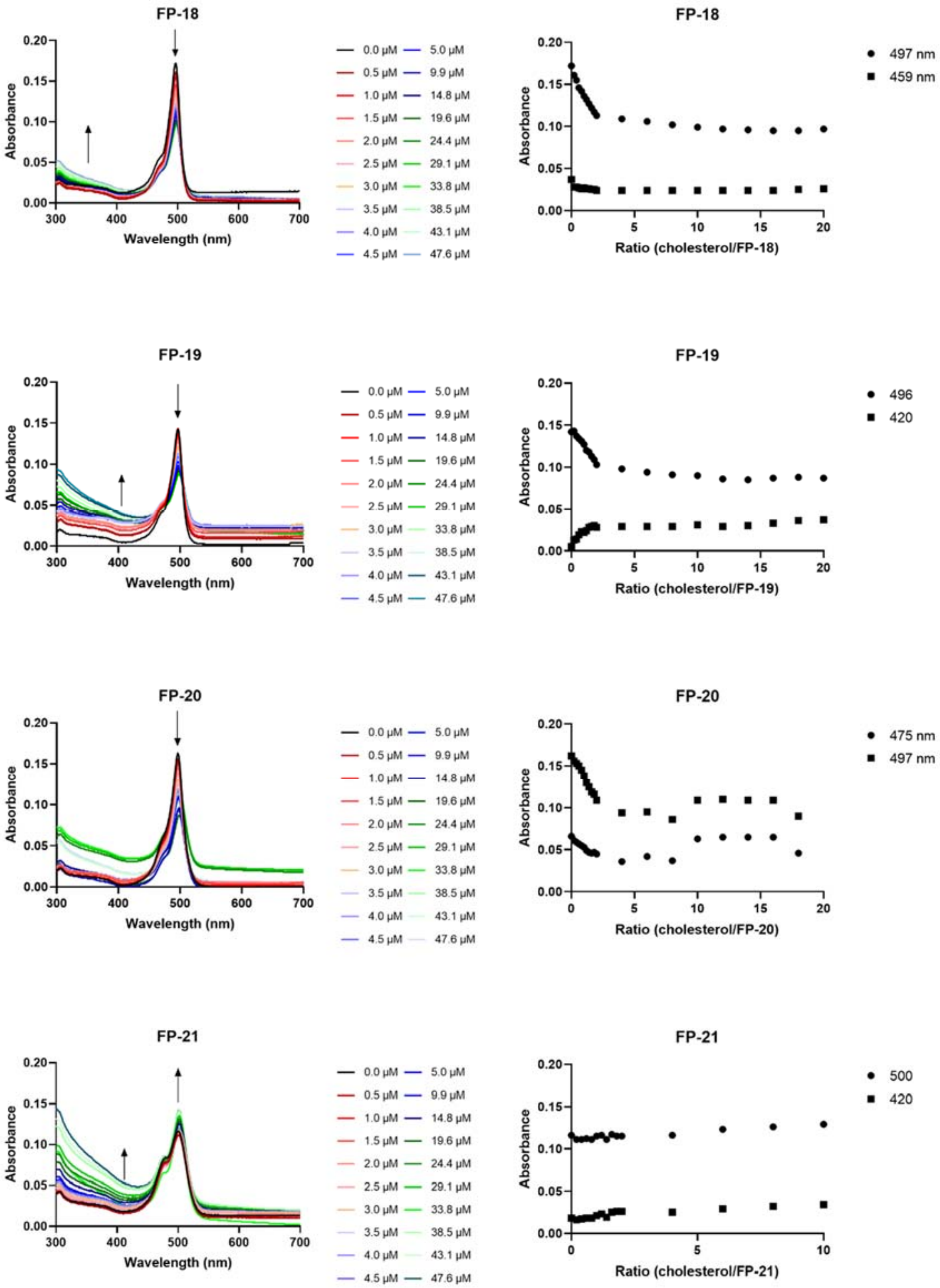

Figure S7: Interaction of **FP-5** and **FP-15** – **FP-21** probes with cholesterol measured by UV-VIS and fluorescence spectroscopy. Titration of probes (0.3 μM) with increasing concentrations of cholesterol (0 – 47.6 μM) in 10% DMSO (left panels). The absorbance changes measured at various wavelengths (right panels).

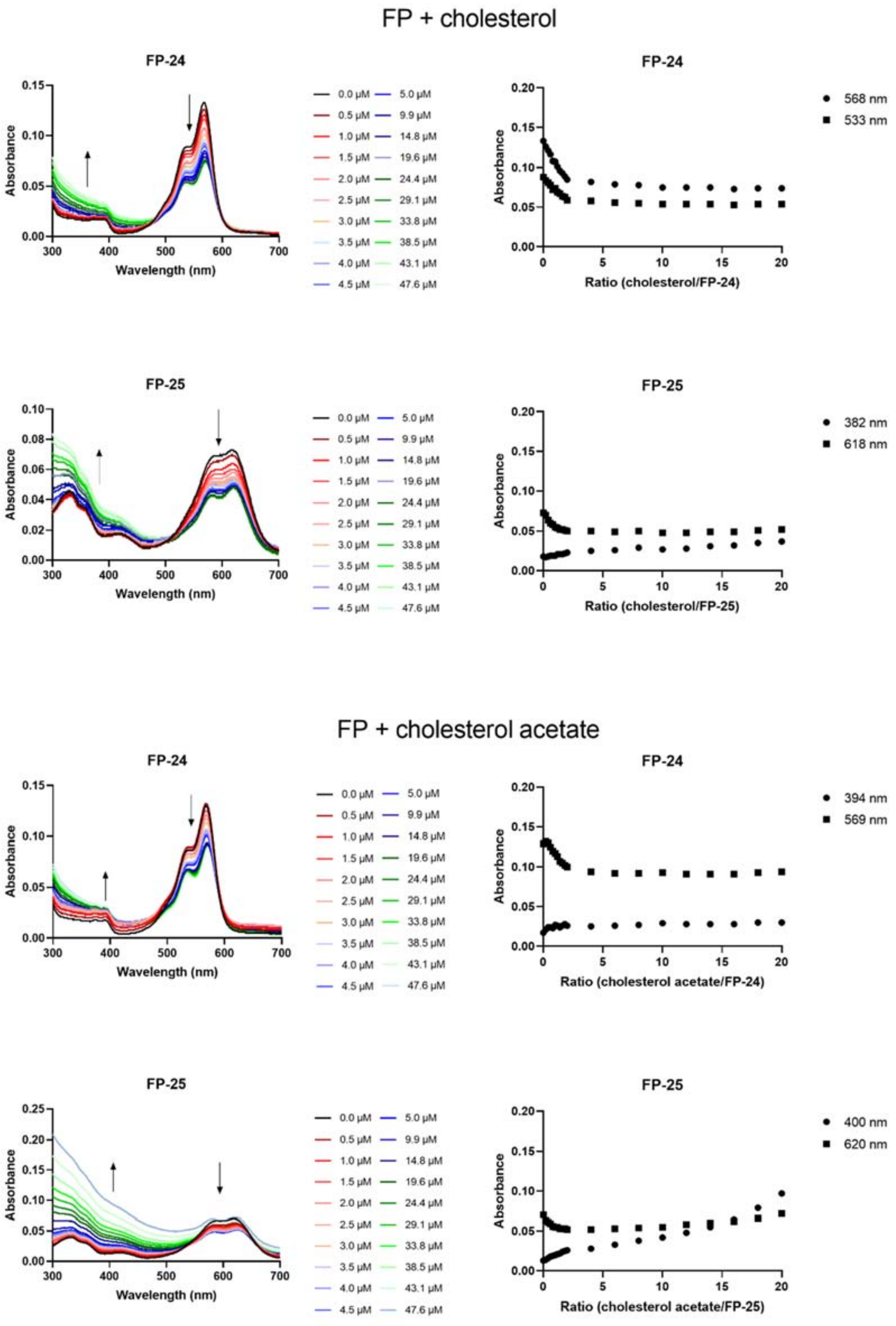

Figure S8. Interaction of **FP-24**, **FP-25** with cholesterol and cholesterol acetate measured by UV-VIS spectroscopy. Titration of probes (0.3 μM) with increasing concentrations of cholesterol and cholesteryl acetate (0 – 47.6 μM) in 10% DMSO (left panels). The absorbance changes measured at various wavelengths (right panels).

Table S3. Value of stability constants and stoichiometry of probe complex with analytes in aqueous medium H_2O-DMSO, 9/1 (*v/v*, resp.).

**Log k_s**				
Probes **A**	Spacer	Tested analyte	Complex stoichiometry (probe : cholesterol)	
			1:1	2:1
**FP-15**	C-1	Cholesterol	7.0247	7.1023
**FP-16**	C-3	Cholesterol	2.4233	8.3080
**FP-5**	C-4	Cholesterol	6.0659	10.5986
**FP-17**	C-5	Cholesterol	5.3848	4.7651
**FP-18**	C-7	Cholesterol	6.2577	6.1532
**FP-19**	C-9	Cholesterol	6.1694	5.6325
**FP-20**	C-11	Cholesterol	5.1531	6.3258
**FP-21**	C-15	Cholesterol	1.4499	11.0583
Probes **B**	Spacer	Tested analyte	Complex stoichiometry (probe : analyte)	
			1:1	2:1
**FP-24**	C-4	Cholesterol	6.1878	5.4445
		Cholesteryl acetate	6.4464	5.3234
**FP-25**	C-4	Cholesterol	3.9343	7.1597
		Cholesteryl acetate	1.3349	9.0273

4. Cell imaging

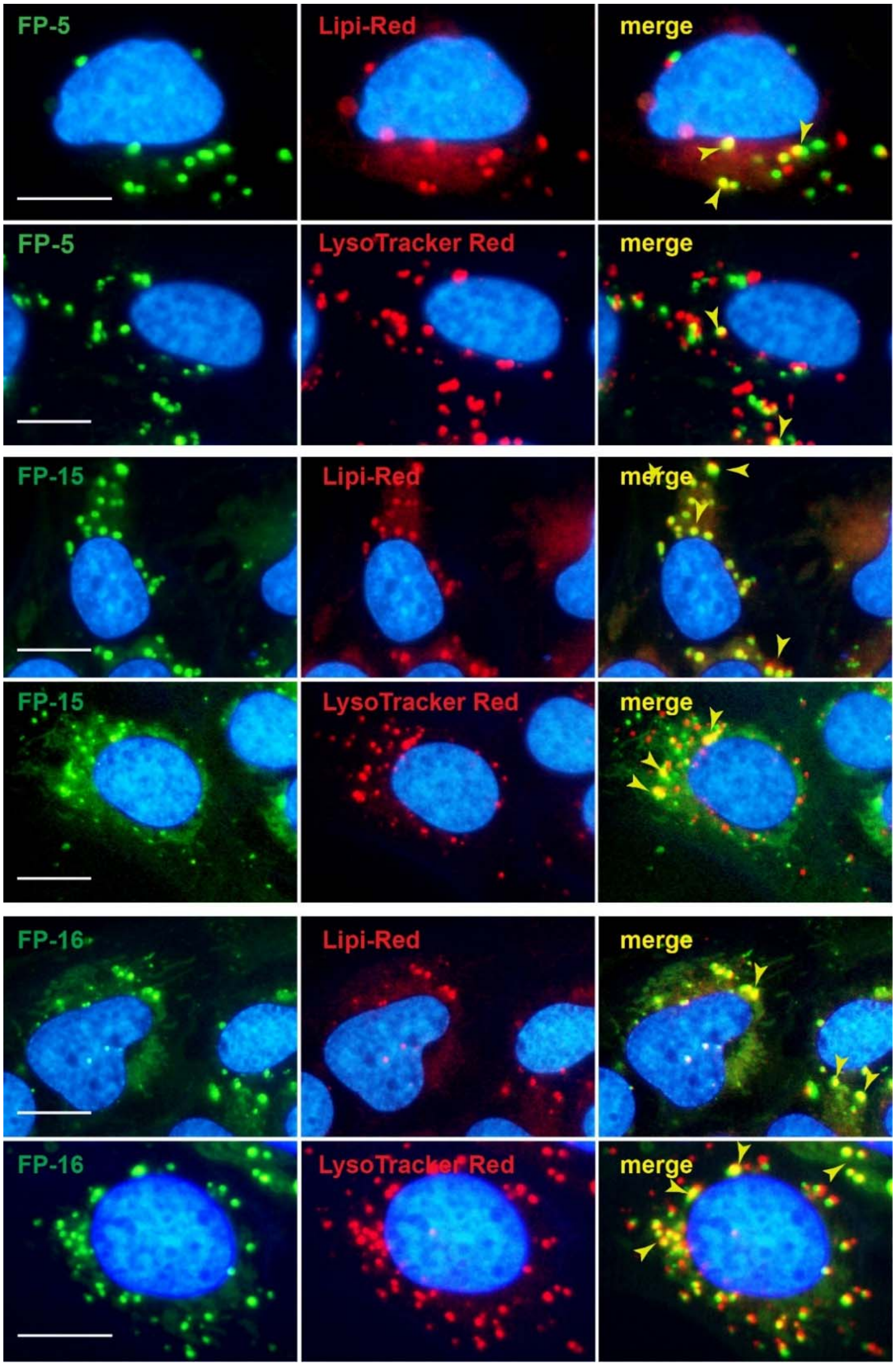

Figure S9. Co-localization of probes **FP-5**, **FP-15**, and **FP-16** with organelle specific markers Lipi-Red or LysoTracker-Red. U-2 OS cells were loaded with probes at 200 nM concentration in serum free FluoroBright medium for 24 h and then co-cultivated for 30 min with 1 μM Lipi Red or 0.1 μM LysoTracker- Red. For the last 5 min, dye Hoechst 33342 was added to stain nuclei. Pictures were taken at identical settings. Co-localization with markers Lipi-Red for lipid droplets (LD) or LysoTracker-Red for lysosomes, indicated by yellow/orange color, was relatively high. Scale bar 10 μm.

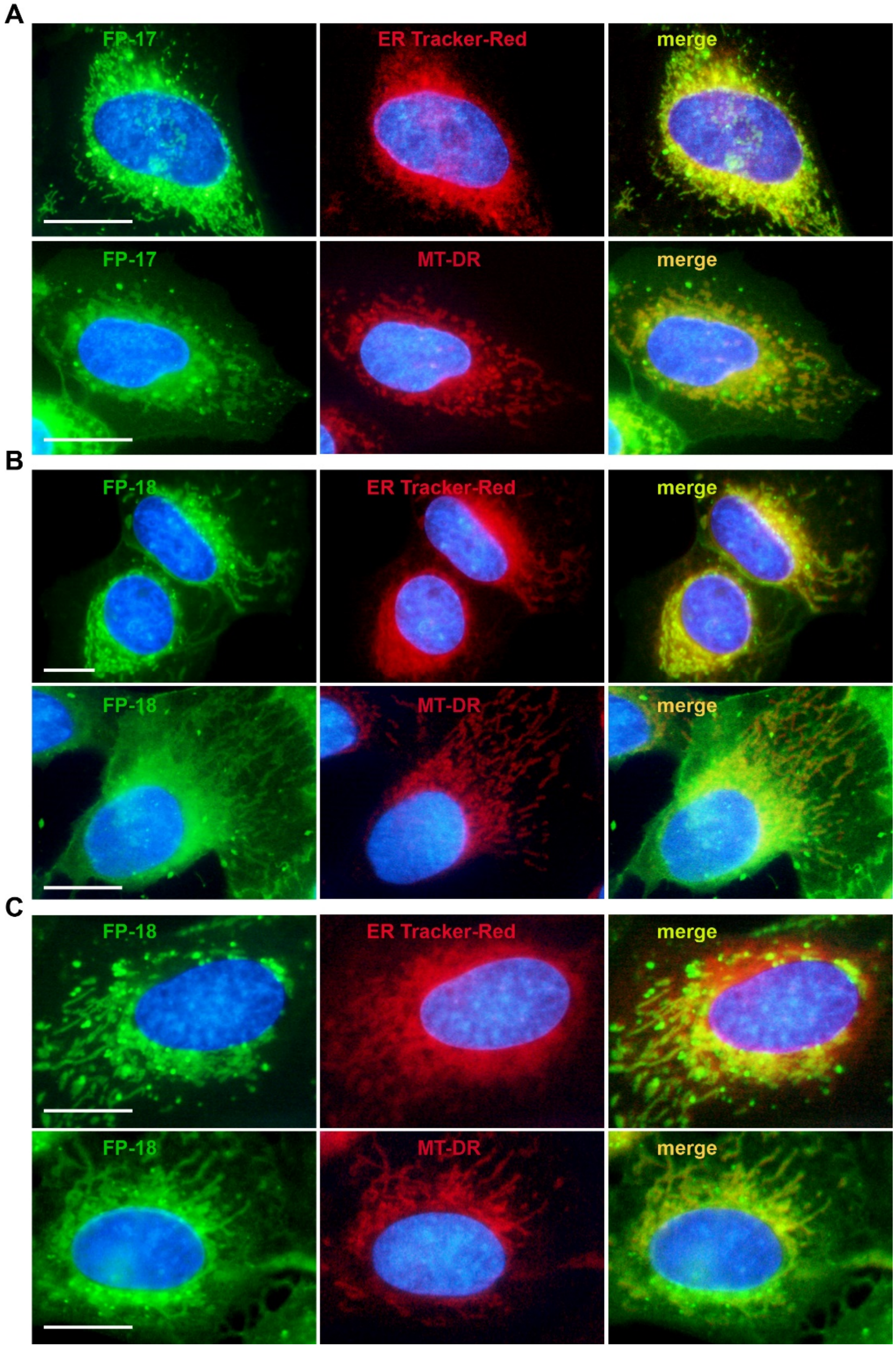

Figure S10. Co-localization of probes **FP-17** and **FP-18** with organelle specific markers ER-Tracker Red and MitoTracker Deep Red. U-2 OS cells were loaded with probes at 200 nM concentration in FluoroBright medium containing 5% LPDS and co-stained with organelle markers. (**A**) Cells labelled with **FP-17** probe for 1 h and then with 1 μM ER-Tacker-Red or 80 nM MitoTracker-Deep Red (MT-DR) for additional 30 min. (**B**) Cells labelled with **FP-18** probe for 1h ,(**C**) cells labelled with **FP-18** probe for 24 h and then with 1 μM ER-Tacker-Red or 80 nM MitoTracker-Deep Red (MT-DR) for additional 30 min. Co-localization, indicated by yellow/orange color, was relatively high. Scale bar 10 μm.

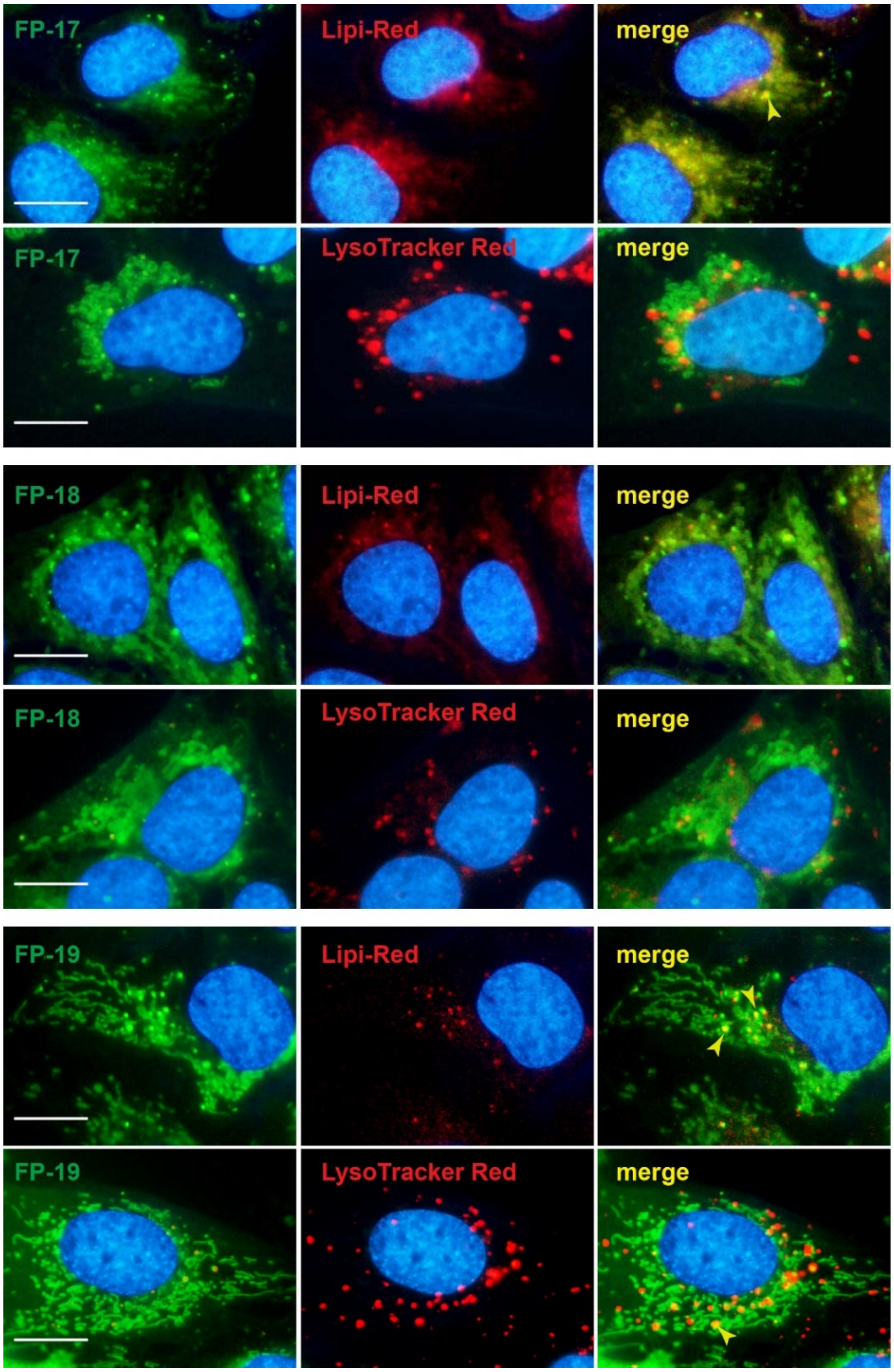

Figure S11. Co-localization of probes **FP-17**, **FP-18**, and **FP-19** with organelle specific markers Lipi-Red or LysoTracker-Red. U-2 OS cells were loaded with probes at 200nM concentration in FluoroBright medium for 24 h and then co-cultivated for 30 min with 1 μM Lipi-Red or 0.1 μM LysoTracker-Red. For the last 5 min, dye Hoechst 33342 was added to stain nuclei. Pictures were taken at identical settings. Co-localization with markers Lipi-Red for lipid droplets (LD) or LysoTracker-Red for lysosomes, indicated by yellow/orange color, was rather low. Scale bar 10 μm.

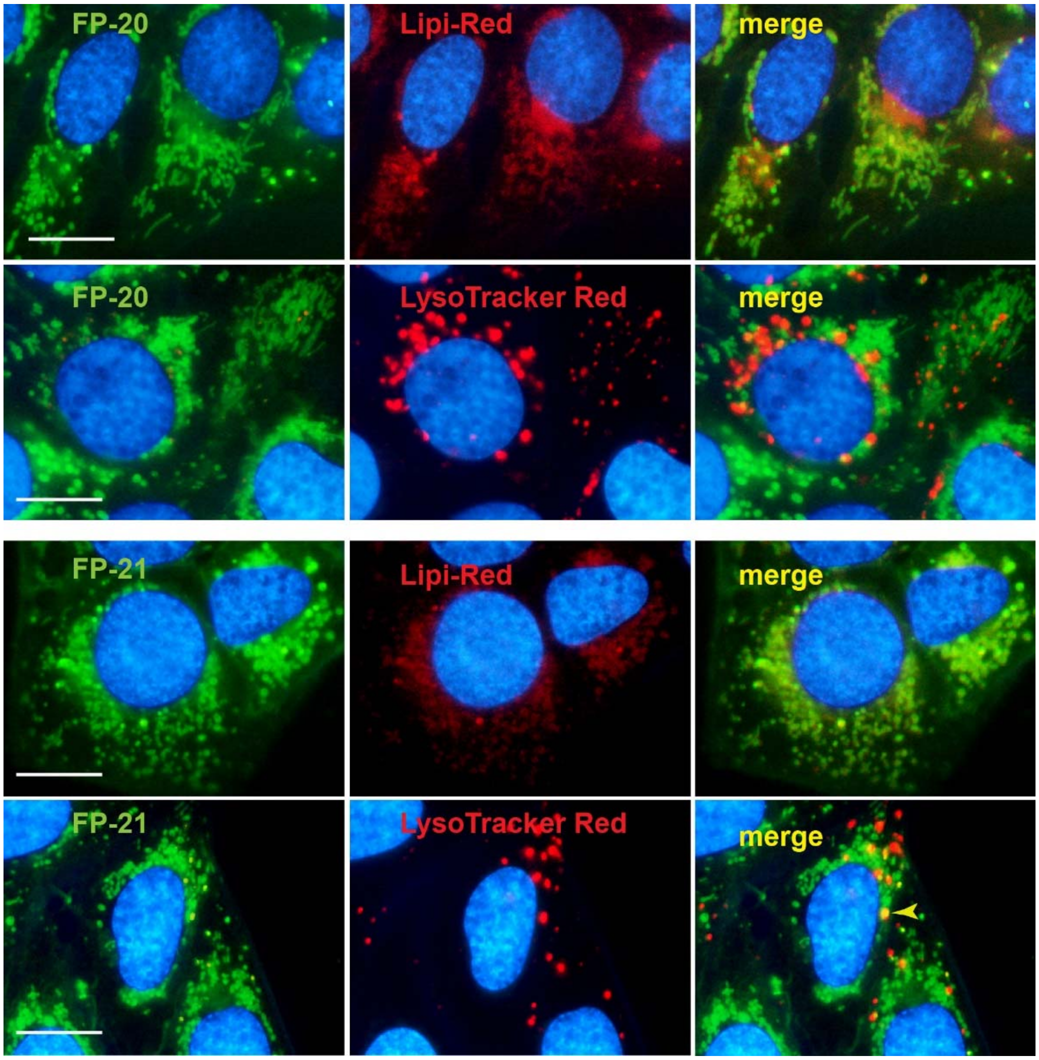

Figure S12. Co-localization of probes **FP-20**, and **FP-21** with organelle specific markers Lipi-Red or LysoTracker-Red. U-2 OS cells were loaded with probes at 200 nM concentration in FluoroBright medium for 24 h and then co-cultivated for 30 min with 1 μM Lipi-Red or 0.1 μM LysoTracker-Red. For the last 5 min, dye Hoechst 33342 was added to stain nuclei. Pictures were taken at identical settings. Co-localization with markers Lipi-Red for lipid droplets (LD) or LysoTracker-Red for lysosomes, indicated by yellow/orange color (shown by arrows), was low. Scale bar 10 μm.

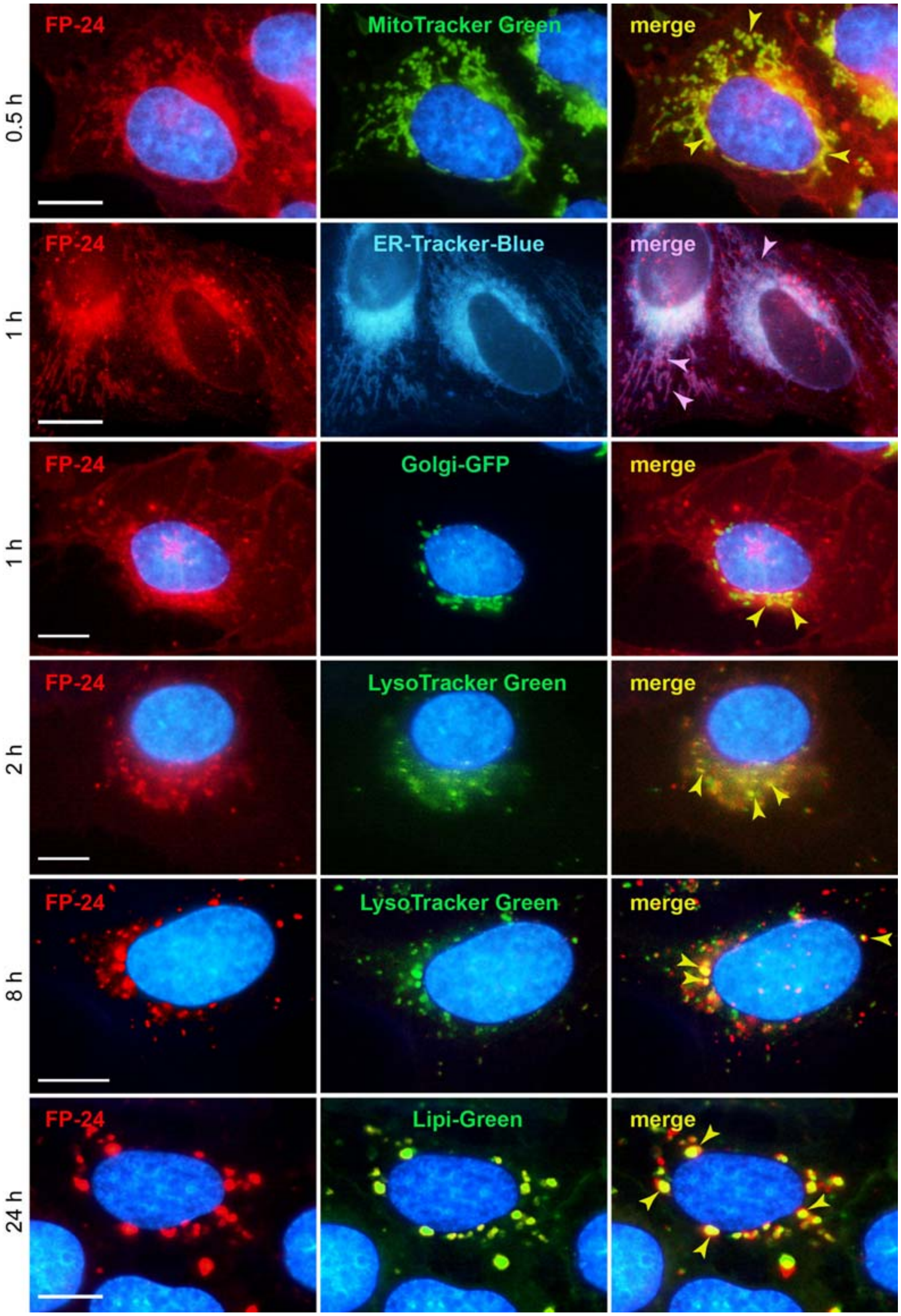

Figure S13. Trafficking of **FP-24** probe in U-2 OS cells. Probe **FP-24** at 50 nM concentration was added to FluoroBright medium for indicated time and subsequently co-cultivated for 30 min with organelle specific markers MitoTracker-Green, LysoTracker-Green or Lipi-Green. For detection of Golgi apparatus, cells were firstly labelled with Cell Light Golgi-GFP and then exposed to **FP-24** for 1 h. For the last 5 min, dye Hoechst 33342 was added to stain nuclei. Co-localization with cell organelle markers (yellow or pink-violet) are shown by arrows. Scale bar 10 μm.

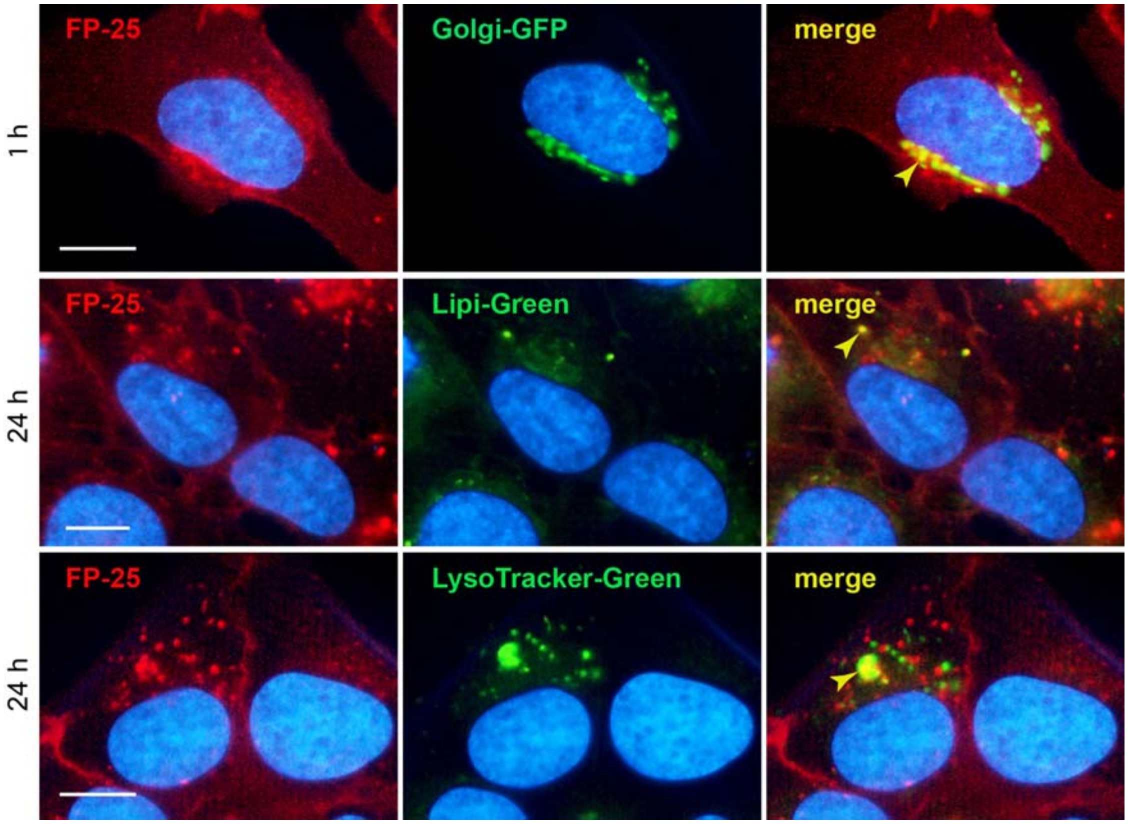

Figure S14. Trafficking of **FP-25** probe in cells. Probe **FP-25** at 200 nM concentration was added to FluoroBright medium for indicated time and subsequently co-cultivated for 30 min with organelle specific markers LysoTracker-Green or Lipi-Green. For detection of Golgi apparatus, cells were firstly labelled with CellLight Golgi-GFP and then exposed to **FP-25** for 1 h. For the last 5 min, dye Hoechst 33342 was added to stain nuclei. Colocalization with cell organelle markers (yellow) are shown by arrows. Scale bar 10 μm.

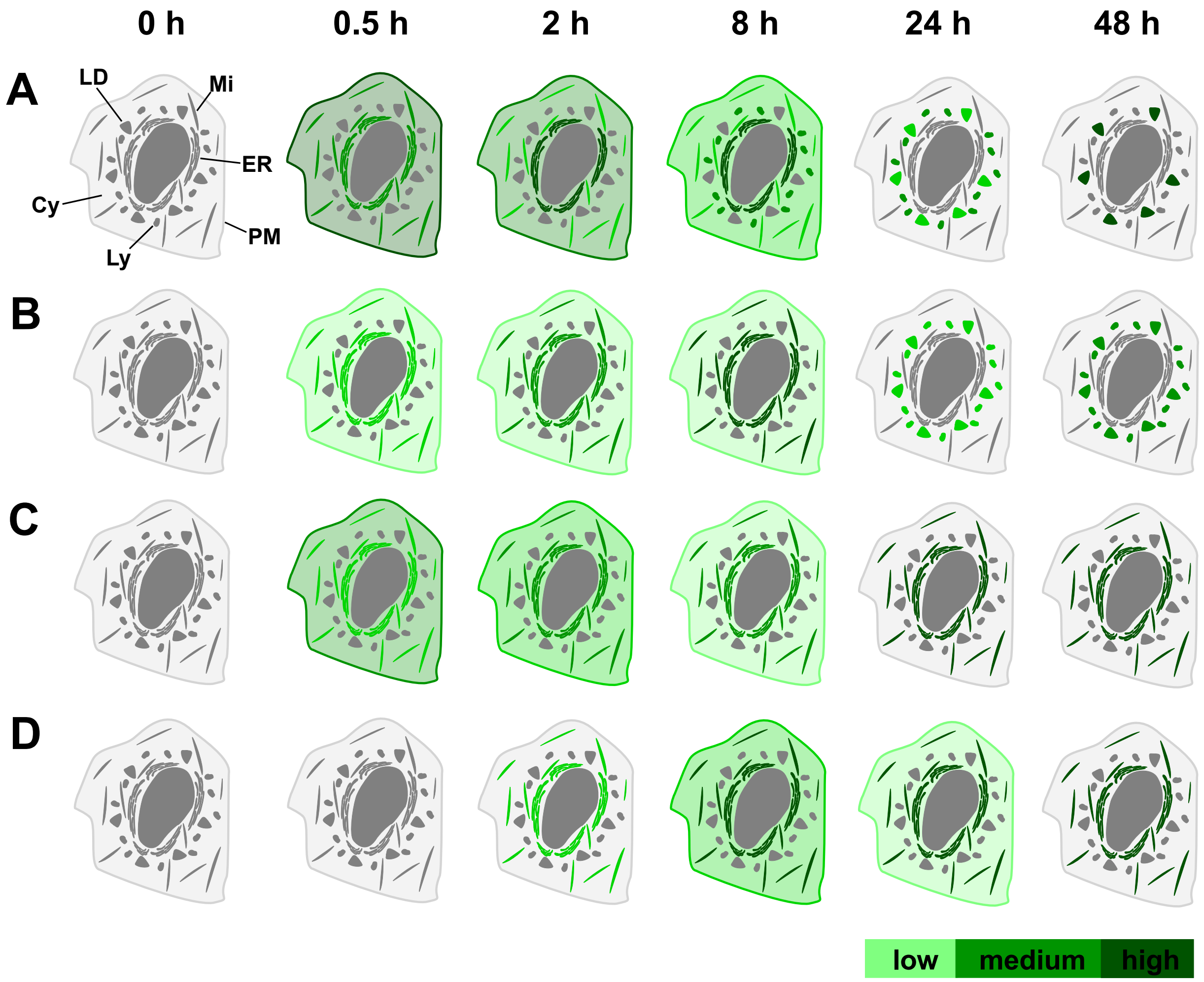

Figure S15. Schematic distribution of **FP-5** (A), **FP-16**, **FP-17** (B), **FP-18**, **FP-1**9 (C) and **FP-20** (D) signal in U2-OS cells.

5. References

1. Kralova, J. *et al.* Heterocyclic sterol probes for live monitoring of sterol trafficking and lysosomal storage disorders. *Sci Rep* **8**, 14428, doi:10.1038/s41598-018-32776-6 (2018).
2. Li, Z., Mintzer, E. & Bittman, R. First synthesis of free cholesterol-BODIPY conjugates. *J Org Chem* **71**, 1718-1721, doi:10.1021/jo052029x (2006).
3. Holtta-Vuori, M. *et al.* BODIPY-cholesterol: a new tool to visualize sterol trafficking in living cells and organisms. *Traffic* **9**, 1839-1849, doi:10.1111/j.1600-0854.2008.00801.x (2008).
4. Solanko, L. M. *et al.* Membrane orientation and lateral diffusion of BODIPY-cholesterol as a function of probe structure. *Biophys J* **105**, 2082-2092, doi:10.1016/j.bpj.2013.09.031 (2013).
5. Liu, Z. *et al.* Synthesis of cholesterol analogues bearing BODIPY fluorophores by Suzuki or Liebeskind-Srogl cross-coupling and evaluation of their potential for visualization of cholesterol pools. *Chembiochem* **15**, 2087-2096, doi:10.1002/cbic.201402042 (2014).
6. Sazonovas, A., Japertas, P. & Didziapetris, R. Estimation of reliability of predictions and model applicability domain evaluation in the analysis of acute toxicity (LD50). *SAR QSAR Environ Res* **21**, 127-148, doi:10.1080/10629360903568671 (2010).

s25
